# Khartoum War's echoes in oil and energy sectors: Economic and environmental implications for Sudan and South Sudan

**DOI:** 10.1016/j.heliyon.2024.e34739

**Published:** 2024-07-23

**Authors:** Mohamed Elnourani, Hamid Suliman Hamid Elhag, Waleed Isameldin Alasad, Mohamed Nasr Bashier

**Affiliations:** a Department of Social Sciences, Technology and Arts, Luleå University of Technology, 971 87, Luleå, Sweden; b Department of Mechanical Engineering, Aalto University ,P.O. Box 11000, Espoo, Finland; c Napesco Petroleum, Alrehab group 130, building 21, New Cairo, Egypt; d Federal Energy, 6th Floor, Business Center, The Meydan Hotel Grandstand, Meydan Road, Nad Al Sheba, Dubai, United Arab Emirates

**Keywords:** Sudan, South Sudan, Petroleum, Khartoum's War, African development, Geopolitical conflicts, Oil and gas sector, Post-conflict recovery, Energy Security, Environmental Degradation, Energy Supply Chain

## Abstract

The energy sector is a main driver of African growth, particularly in regions with geopolitical conflicts like Sudan and South Sudan. The oil and gas industry notably influences these regions' economy, politics, humanitarian situation, and social stability. This study seeks to investigate how the Khartoum War affected the energy sector of both Sudan and South Sudan, particularly looking at the disruptions caused by recent conflicts and their impact on oil production, economic stability, and environmental conditions. The study employs a multi-disciplinary approach, utilising different sources such as regional legal agreements, government reports, academic articles, and press releases from international organisations. The key methodology includes qualitative analysis of several documents and quantitative assessment of production data and economic reports. The study's key findings show a significant decline in oil production and transportation due to the shutdown of key oilfields and pipelines, intensifying economic and humanitarian crises. Additionally, the damage to oil infrastructure has presented serious environmental risks, highlighting the delicate balance between resource management and regional stability. In conclusion, the study's findings underscore the intense impact of the Khartoum War on the energy sector of Sudan and South Sudan, and the urgent need for policy recommendations to mitigate these effects and foster sustainable development.

## 1 Background

The energy sector is essential for African development, driving industrialisation, supporting households, and connecting transportation networks. This importance is especially evident in regions with geopolitical conflicts, where energy resources can be sources of conflict, causing supply disruptions, price instability, and broader economic risks [1,2]. An important case can be seen in Sudan and South Sudan, where oil plays a crucial role in the economy and contributes to ongoing conflicts and political instability. Literature shows that the abundance of oil in these areas has brought both positive and negative outcomes, creating economic growth while also intensifying tensions and shaping governance and conflict patterns [3]. This demonstrates the intricate relationship between natural resources and political stability in Africa [4,5].

Sudan is confronted with substantial challenges caused by environmental consequences, mostly resulting from climatic fluctuations and environmental stress. Since the 1970s, the Sudan-Sahel area has seen significant climate changes characterised by reduced rainfall rates and increased temperatures [6,7]. Environmental degradation involves soil erosion, depletion of water resources, and loss of vegetation, which significantly affect agricultural productivity and livelihoods [8]. These climatic shifts, exacerbated by human activities, create a complex interplay between environmental stress and socio-economic conditions, necessitating adaptive measures to manage natural resources effectively [1,9]. Understanding these dynamics is crucial for developing effective energy policies that can mitigate environmental degradation while promoting sustainable development. The reliance on biomass for energy in Sudan further strains the environment, as deforestation and land degradation reduce the availability of fuelwood, leading to a vicious cycle of environmental degradation and energy scarcity [10]. Addressing the environmental implications requires integrated strategies that consider sustainable energy solutions to mitigate the adverse effects and promote resilience [11]. The United Nations Environment Programme (UNEP) highlights the urgency of integrating environmental considerations into national energy policies to ensure sustainable development and climate resilience in Sudan and South Sudan, among other countries experiencing conflicts [12]. Furthermore, the United Nations Development Programme (UNDP) stresses the need for innovative approaches to energy production that reduce environmental impact while meeting the growing demand [7].

Following the separation of South Sudan from Sudan in 2011, both nations have continued to grapple with the dual challenge of managing their oil wealth and navigating ongoing political conflicts. The fragility of this balance was highlighted by the outbreak of renewed conflict in Sudan in April 2023 [[13], [14], [15]], which severely impacted oil production operations, infrastructure, and the broader economic landscapes of both countries. This conflict not only disrupted oil supply chains but also intensified regional and international geopolitical tensions, demonstrating the high stakes associated with energy security in the region [16,17].

This article explores the complex relationship between the oil industry and the current geopolitical situation in Sudan and South Sudan, with a focus on the disruptions caused by the 2023 conflict. It assesses the direct impacts on oil production facilities, including the shutdown of major oilfields, pipelines, the consequent economic implications, and the broader humanitarian crises that these events have exacerbated. By utilising the “Oil and Related Economic Matters” agreement [18], production reports from both nations, press releases from NGOs, and incorporating insights from recent academic and policy analyses, the study aims to offer a nuanced understanding of the ongoing challenges and potential pathways forward. This comprehensive approach is innovative as it integrates economic, legal, technical, and geopolitical dimensions, augmented by satellite imaging, to create an overarching understanding of the conflict. This methodology not only highlights the immediate and long-term impacts on the energy sector but also lays the foundation for future focused studies on post-conflict scenarios in Sudan and South Sudan.

The contributions of this research include a detailed analysis of the energy-conflict nexus in Sudan and South Sudan, highlighting innovative approaches to mitigating environmental impacts and promoting energy security amidst political instability. By identifying key innovations in energy production and exploring their potential applications in the Sudanese context, this study aims to inform policy interventions that foster stability and sustainable development in a region critically dependent on its energy sector. This study underscores the critical need for robust international engagement and innovative policy interventions by integrating up-to-date legal frameworks, current economic data, and geopolitical insights. These are essential not only to stabilise the current situation but also to lay the groundwork for sustainable development and peace in both Sudan and South Sudan.

## 2 Methodology

This study adopts a multi-disciplinary approach to explore the complex dynamics between geopolitical conflict and the oil sector in Sudan and South Sudan, particularly focusing on the disruptions caused by the 2023 conflict. The methodology is structured as follows.

• Data Collection:

Primary Sources: The analysis is primarily based on the “Oil and Related Economic Matters” agreement between Sudan and South Sudan [18], which provides a legal framework and context for the study. Additional primary data were collected from recent production reports from Sudan and South Sudan [19], press releases, and direct statements from key stakeholders within both governments and oil companies operating in the region. Moreover, the Google Earth Engine GIS platform is used to create aerial images and maps of the current damage to oil and gas facilities.

Secondary Sources: Includes academic articles, policy papers, and news articles, particularly those that focus on the economic and political implications of oil in Sudan and South Sudan. The work by Le Billon and Savage [20] “Binding pipelines? Oil, armed conflicts, and economic rationales for peace in the two Sudans” was instrumental in providing a theoretical background on the economic motivations behind peace and conflict in oil-rich regions.

• Data Analysis

Qualitative Analysis: This involved a thematic analysis of content from both primary and secondary sources to identify and categorise the impacts of conflict on the oil sector based on the three triple bottom lines of sustainability in order to capture the direct and indirect effects both in ecological and social levels also as suggested by Schillinger et al. [21].

Quantitative Analysis: Utilized statistical data from the South Sudanese governmental website and other relevant economic reports to quantify the economic impact of the conflict on oil production and revenue losses. This analysis helps in establishing a clear link between conflict events and economic outcomes.

## 3 Theoretical background

### 3.1 Relationship between energy, development and environment

The complex relationship between energy consumption and environmental sustainability has been the focus of substantial scholarly study, with a growing body of literature shed light on the delicate processes involved. Energy consumption is a prerequisite for economic growth, but it is also a major cause of environmental deterioration, especially due to carbon emissions [[22], [23], [24], [25]]. Consequently, achieving a balance between energy use and environmental preservation is paramount. The United Nations Environment Assembly underscores that sustainable economic growth can be attained through the efficient utilisation of energy and a transition towards renewable energy sources, which significantly reduce carbon emissions [26,27]. To achieve such balance, environmental policy measures are critical to enhancing energy efficiency and mitigating environmental impacts. Innovations in environmental technology, such as advancements in renewable energy technologies, play a key role in reducing carbon emissions and ensuring energy efficiency in production and consumption [23,28].

### 3.2 The impact of wars on the energy sector, environment, and economy

Wars have profound environmental impacts beyond simply the immediate human dimensions in which they occur, especially in oil and gas-rich regions. Recent research indicates that military actions aimed at energy resources have led to widespread damage to ecosystems, air quality and soil conservation. Severe ecosystem fragmentation occurs because of military-related activities like oil spills, blowing up pipelines or clearing forests to provide space for military camps [29,30]. Lacher and Kumetat [31] emphasise the long-term environmental consequences of military conflicts, including soil degradation and loss of biodiversity, which persist long after hostilities cease. Looking at particular cases, Wars have severely affected the energy sector, environment, and economy, as seen in the situation in Iraq, Kuwait [32], and Ukraine [33]. The energy sector in Iraq was destabilized after the United States invaded it in 2003; even though it is the second biggest oil producer in OPEC, there are still security challenges regarding its energy, and investing in it is a risk due to corruption and political instability [34]. Kuwait's Gulf War lasted from 1990 to 1991 and involved significant pollution problems due to oil spills and fires that affected air and ground quality. Similarly, the Russian invasion of Ukraine in 2022 resulted in an enormous energy deficiency in the central region of Europe, prompting the swift adoption of alternative energy sources and liquefied natural gas imports from other suppliers [35]. Amidst all of these wars, the intersections of struggles related to interruption in the operations of power plants, exhausting of nature and rolling financial setbacks are pointing to the significance of regional and international rebuilding plans in the aftermath of armed conflicts, which look to ensuring an enduring reconstruction to secure energy production and distribution [5,36].

### 3.3 Oil and gas facilities in Sudan

**Fig. 1:**
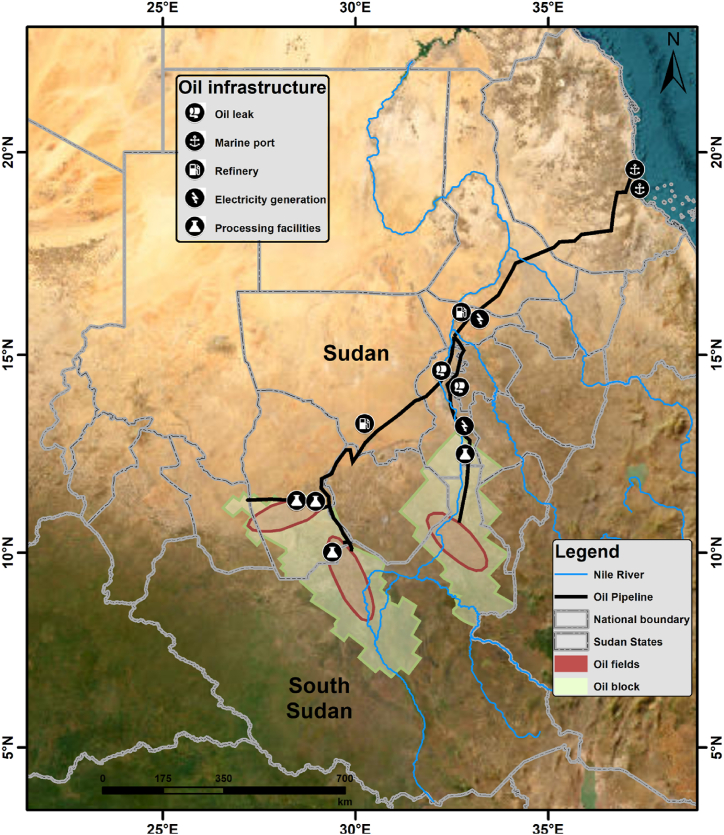
A map of major oil and energy infrastructure affected by the ongoing War in Sudan. Note: The above map provides an approximate illustration of the major oil and energy infrastructure impacted by the ongoing conflict in Sudan. Please note that The PETCO and BAPCO pipelines are represented as a single line from Khartoum to PortSudan, and the Petro-Energy and PECTO pipeline lines from Obeid to Khartoum are also shown as a single line. For detailed information about these specific lines, please refer to Table 1.

The oil and gas industry in Sudan involves an integrated system of exploration, extraction, processing, and transportation facilities (See Fig. 1). This includes oil wells, processing facilities, pipelines, refineries, Marin terminals and depot facilities. This infrastructure is fundamental for a country's economic growth, meeting energy demands, and earning revenue from exports [16,[37], [38], [39], [40]].

#### 3.3.1 Upstream (active companies, fields)

Heglig Oil Field: As one of Sudan's largest remaining oil fields, Heglig has an essential role in the country's oil production. Located near the border with South Sudan, it has been a focal point of conflict between the two nations. The field is characterized by mature oil wells, necessitating enhanced oil recovery techniques to maintain production levels, Bamboo Oil Field: Located in the Muglad Basin, this field is less developed compared to Heglig but holds significant potential for future development with adequate investment and technology. Moreover, within Sudanese borders with smaller capacity, there are Balila, Sharif and Alrawaat Oil Fields located in Muglad Basin. These fields contain complex networks of central processing units (CPF), Field Production Facility (FPF), Oil Gathering Manifolds (OGM), oil wells, storage tanks, and water processing facilities [41]. See (Fig. 1).

#### 3.3.2 Pipelines

The capacity and operational efficiency of Sudan's pipeline networks are crucial for both the import and export-oriented sectors of the national economy. Although there is an extensive network of pipelines connecting oil fields, strategic storage stations across the country, and distributing oil products to different regions, there are three main pipelines that have the highest economic value (see Table 1). Greater Nile Oil Pipeline: This 1500 km pipeline is crucial for transporting crude oil from the southern oil fields to the northern export terminal at Port Sudan on the Red Sea. The pipeline's capacity and operational efficiency are pivotal for the export-oriented sector of Sudan's oil economy. BAPCO Oil Pipeline: Operated by BAPCO Company, this pipeline spans 1368 km from the Upper Nile State in South Sudan to the Red Sea in Sudan. Petro-energy Oil Pipeline: the line is 24 inch pipeline operated by Petro-energy company and it connects AlFula CPF to Khartoum refinery. Moreover, there are other pipelines for importing oil products with smaller capacities. 12 inches for importing gas oil while 8 inches for importing Mogas both are operated by the Sudanese Petroleum Pipeline company [18,20,39,41,42]

**Table 1:** Sudan crude oil transportation system (see Fig. 1).

Item	Petrolines for crude oil pipeline (PETCO)	Bashir Pipeline Company (BAPCO)	Petro Energy (PE)
Pipeline Route	Heglig - Bashaier-1	Falouge - Bashaier-2	AlFula - Khartoum
Capacity, bbl/day	450000	500000	200000
Length, Km	1610	1500	720
Diameter, inch	28	32	24
Crude	Nile blend	Dar crude	Fula Crude
Operator	PETCO	BAPCO	Petro Energy
Owner	Sudan Government	Sudan Government	Petro Energy (PE)
Pump station	6	6	5
Pump station location	Heglig, Eldalange, Um saiala, Eljaili, Elhody and Haya	Falouge, Algabalyn, Naiema, eleliafone, Gabal Um ali and	Fula, Auzabad, Obaied, Um saiala and west of Omdurman

**Table 2:** Sudan Oil Products Transportation System) based on the official website of the Ministry of Petroleum.

Item	8 Inches Pipeline	12 Inches Pipeline
Pipeline Route	Portsudan-Shagra	Portsudan = Algaily
Commissioning Date	1978	2005
Length, Km	815	741
Diameter, inch	8	12
Products	Mogas/Gasoil/Keronene	Mogas/Gasoil/Keronene
Operator	Sudanese Petroleum Pipelines Company	Sudanese Petroleum Pipelines Company
Owner	Sudan Government	Sudan Government
Pump station	6	2
Pump station location	Portsudan, Erkawit, Haya, Elrogel. Atbra, Algaily + Shagra (Terminal)	Algaily, Portsudan

#### 3.3.3 Refineries

Khartoum Refinery: Situated near Sudan's capital, this refinery has a capacity of approximately 100,000 barrels per day. It primarily processes crude oil into petroleum products for domestic consumption and some exports. The refinery is a joint venture between Sudan and China, reflecting the significant foreign investment in Sudan's oil sector, Moreover, a Garri thermal power plants complex attached to Khartoum Refinery to utilise local resources and reduce fuel transportation costs[[43], [44], [45]]. Moreover, Khartoum Refinery also supports the implementation of an oil product supply agreement with Ethiopia, further emphasizing their strategic importance in regional energy security (IMF, 2020; 10.13039/501100018760OEC, 2022). The Obayed Refinery in Sudan is a relatively smaller oil processing facility compared to Khartoum Refinery, which produces 10,000 to 15,000 barrels per day. It focuses primarily on meeting local demand within the region, processing crude oil into various petroleum products like Naphtha, Kerosene, Gas oil, and Furnace oil [46].

#### 3.3.4 Marine terminals

There are three marine terminals in Sudan for oil products and crude. These include Bashayer, which is the endpoint of the PETCO pipeline, Bashayer 2, which is the endpoint for the BAPCO pipeline, and AL Khair terminal, which is utilized for importing oil products for local consumption and ethanol imports.

PortSudan Terminal: This terminal is the endpoint of the Greater Nile Oil Pipeline, where the oil is stored and loaded onto tankers for export. The facility's strategic importance cannot be understated, as it is the primary maritime outlet for Sudanese oil exports [39] (see Table 2).

#### 3.3.5 Refined products strategic depots in Sudan

The oil *products* depots in Sudan play a crucial role in supporting various sectors such as agriculture, aviation, and electricity generation. They also help in ensuring stable energy supplies locally and regionally. These depots are essential for Sudan's economy, especially during unpredictable global market changes and political tensions in the region. The information in Table 3 gives a thorough overview of the oil products stored at various strategic depots in important urban and industrial areas such as Portsudan, Khartoum, and the White Nile regions. The Old and New Strategic Depots in Portsudan play a major role in facilitating transportation between the main port and other parts of the country. Additionally, the Gaili Strategic Depot in Khartoum North is crucial for providing energy, as it holds the biggest reserves of gasoil and LPG, which are essential for powering cities and industries in the area [47].

**Table 3:** Overview of strategic oil depot capacities and distribution in Sudan in Metric Tons (MT).

Depot Name	Location	Gasoil (MT)	Mogas (MT)	LPG (MT)	Jet-A1 (MT)
**Old Strategic Depot**	Portsudan	33200	58400	1200	0
**New Strategic depot**	Portsudan	66400	36500	10000	15600
**Gaili Strategic depot**	Khartoum North	166000	36500	21000	15600
**Shagra**	Khartoum	61380	38870	0	0
**Rabak Old**	Rabak	5800	0	0	0
**Rabak New**	Rabak	41500	7300	1500	0
**Alhudi**	Atbara	16600	7300	100	0
**Algadarif**	Algadarif	8300	2920	100	0
**Nyala**	Nyala	8300	2920	100	0
**Madani**	Madani	32500	7500	0	0
**Total**		**439980**	**198210**	**34000**	**31200**

### 3.4 International law and protection of resource infrastructure in conflict zones

International law plays a critical role in managing cross-border oil resources and facilities, particularly in contentious regions like Sudan and South Sudan. The principle of permanent sovereignty over natural resources, as enshrined in the United Nations General Assembly Resolution 1803 (XVII) of 1962, establishes that states have the right to control and exploit their resources. This principle, however, often conflicts with the realities of post-colonial state boundaries and internal divisions, leading to disputes over resource ownership and management [48].

During armed conflicts, the protection of energy infrastructure is paramount. International humanitarian law, particularly through the Geneva Conventions and their Additional Protocols, mandates that warring parties protect civilian infrastructure, which includes oil and gas facilities unless used for military purposes. Yet, in conflict zones such as Sudan and South Sudan, these facilities often become targets for military advantage or economic warfare [49]. Moreover, Ajiya [50] and Dobbins Zabyelina and Kustova [51] provide insights into the strategies used by state and non-state actors to secure energy facilities, with applications in both the Middle East and Africa. These studies underscore the complexities of protecting infrastructure in war-torn regions and the potential roles of international peacekeeping forces.

### 3.5 Understanding the sensitivity of oil transportation infrastructure

The oil transportation system, comprising an intricate network of pipelines, pump stations, storage facilities, and heating stations. This network requires continuous management and meticulous supervision to ensure operational integrity and efficiency. This system is highly sensitive to unexpected shutdowns or disturbances, which can significantly disrupt the flow and processing of oil. Pipelines, which are the backbone of oil transport infrastructure, necessitate constant monitoring and maintenance to prevent leaks, ruptures, and blockages. The complexity of these systems stems not only from their physical expansiveness but also from the variety of operational challenges they encounter, including corrosion, mechanical failures, and hydraulic inefficiencies (Smith, 2018; Johnson, 2020). Pump stations and heating stations are equally critical, as they facilitate the movement and viscosity management of the oil, respectively. Any failure in these components can lead to severe operational disruptions. For instance, inadequate heating can cause the thickening of oil, commonly referred to as ‘jellying,’ which can clog pipelines and halt the transportation process, potentially leading to extensive damage to the infrastructure [52]. In the case of Sudan A Research conducted by Mohyaldinn [42] on potential shutdown situations for the Higleig-Portsudan pipeline, which transports Nile Blend, identified a significant risk of solidification occurring at temperatures as mild as 27 °C, particularly following a 12-h shutdown. The study suggested promptly restarting the pipeline to prevent severe harm and solidification. These occurrences have the potential to generate back pressure, ultimately resulting in ruptures and leaks within the pipeline. Storage facilities also play a vital role in maintaining the equilibrium within the transportation system. They buffer the flow of oil between production and demand, thus requiring precise control and regular inspection to avoid overfilling and spills, which pose environmental and operational risks [53,54]. The continuity and effectiveness of maintenance are crucial for preventing and mitigating these risks. Regular and thorough inspections, timely maintenance, and rapid response to operational anomalies are essential to safeguard the infrastructure from both partial and complete failures. Damage to any part of this interconnected system can have cascading effects, impacting not only the site of the incident but also the overall functionality of the oil supply chain [55].

## 4 Khartoum war context

The world does have a legal framework governing parties’ behaviour in war, This is the International Humanitarian Law (IHL), IHL affords special (heightened) protection to certain types of energy infrastructure, notably objects indispensable to the survival of the civilian population, as well as works and installations containing dangerous forces. This special protection applies even in the case where such pieces of energy infrastructure constitute military objectives [56,57], although a robust law exists but the degree of compliance with this law in the ongoing wars around the globe is minimal, infrastructure facilities are targeted by warring parties amplifying the suffering of civilians, this is the case In Sudan as one of the countries encountering ongoing war between Government Armed Forces and the paramilitary Rapid Support Forces. Edem Wosornu, the director of operations at the UN Office for the Coordination for Humanitarian Affairs (OCHA) stated, “*By all measures – the sheer scale of humanitarian needs, the numbers of people displaced and facing hunger – Sudan is one of the worst humanitarian disasters in recent memory*” [58].

Oil industry facilities destruction was a part of the ongoing war intentionally by targeting these facilities directly or indirectly by creating an unsafe environment for workers, unstable supply of needed consumables and logistical issues, and disruption of the communication network, which in turn led to shutdown, looting and theft of the facilities. After the war outbreak in Sudan in April 15, 2023 the Oil Sector was one of the most affected sectors by the war; some producing blocks shut down like block 6 producing about 20 thousands BPD block 17 producing around 2 thousand BPD and block 4 (partially) producing about 5 thousand BPD, the Petro-Energy pipeline shut down (28 inches pipeline 713 Km), the looting of Balela Central Processing Facilities offices furniture, operating vehicles, spare parts, tools and equipment in block 6, Obied refinery, partial destruction of some Khartoum Refinery Facilities and the latest was the shut down of BAPCO Pipeline transporting the Dar Crude oil from South Sudan Block 3 and 7 operated by DPOC which is a consortium that includes Nilepet, China's CNPC and Sinopec and Malaysia's Petronas, with daily production of 95000 BPD, the crude is transported to the Marine Terminal in Bahayer Oil terminal on the Red Sea crossing Sudan territory (32 inches pipeline ∼1600 Km total length).see (Table 1).

**Fig. 2:**
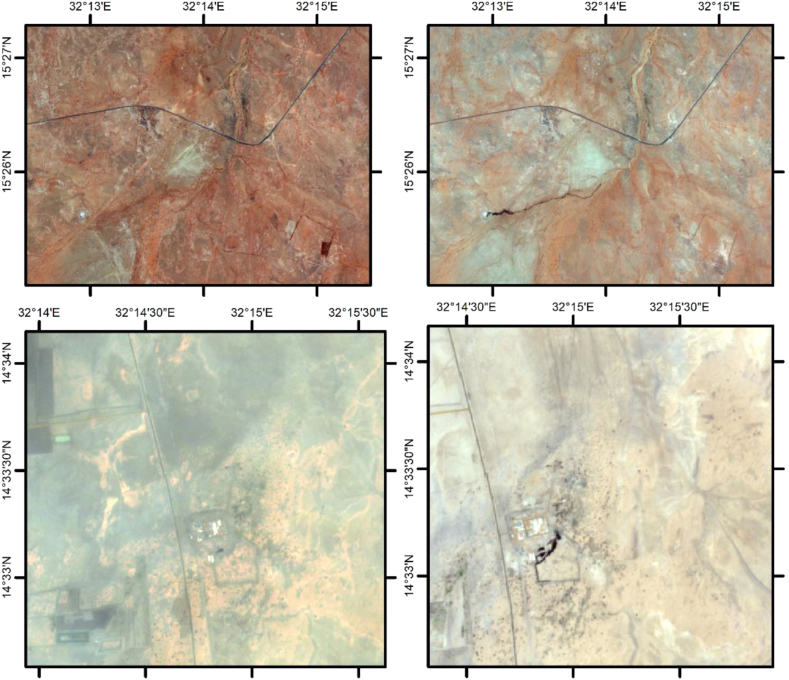
The Map illustrates oil leaks near PETCO Pumpstation No. 4 and BAPCO Pumpstation No. 4. Please refer to Note Below for further details. Note:True Color Composite images were developed by Google Earth Engine (GEE). The images utilize Sentinel-2 data used COPERNICUS/S2 collection, filtered to attain two specific time periods: PETCO pumpstation No 3 April 5, 2023 - April 14, 2023 (Before) and BAPCO pumpstation No 3 April 5, 2024 - April 14, 2024 (After). For both attached images, the B4 (Red), B3 (Green), and B2 (Blue) bands have been chosen to produce True Color composites. The "Before" image uses a Median Composite method and the "After" image uses a Max Composite method.Each image was exported at a scale of 10 meters/ pixel, covering the given zone.

BAPCO pipeline shut down in March 2024 due to diesel shortage in Pump Station 4, which powers the heaters used to in heating crude oil to prevent gelling. The pumped crude without heating from Pumps Station 4 caused the gelling of the crude in the area between Pump Station 4 and Pump Station 5 which in turn created a pressure buildup to higher to the rates exceeding the maximum allowable operating pressure and consequently led to the rupture of the pipeline in the area between pump station 4 and pump station3 as a result Sudan's Ministry of Energy and Petroleum declared a *Force Majeure* (See Fig. 2) stating it is a failure to meet it's obligation in delivering crude oil in and through the BAPCO pipeline to the Bashayer 2 Marine terminal (see Table 1).

## 5 Discussion

### 5.1 General economic impact on Sudan and south Sudan

The current conflict in Sudan has had a far-reaching impact on economic factors, extending beyond the oil industry in Sudan, reflecting the patterns found in the existing literature of conflict zones where the collapse of infrastructure and governance exacerbates economic decline [36,51,56]. The region's economic recovery has been severely hindered by a confluence of crises, aligning with the broader findings of Waheed et al. [25] and Mutumba et al. [22] Mutumba et al. [22] on the impact of external shocks on economic sustainability. These include ongoing conflict, COVID-19 pandemic effects, climate-related disasters, reduced oil production, and global price hikes from events like the war in Ukraine. The recent conflict between Sudan and South Sudan has caused a major economic decline [13]. This is mainly because critical oil production has stopped and there has been damage to infrastructure. Through this chapter we provide a quick assessment of how this has affected both countries economically, looking at revenue loss, infrastructure damage, environmental issues, and the overall impact on society and the economy inline with Lacher and Kumetat [31] exploration of the long-term consequences of military conflicts on economies and environments. The analysis uses data on oil sales, pipeline operations, and costs associated with the conflict to give a detailed view of the financial and social effects caused by the conflict which echoing the findings of Binetti [36] on how conflicts disrupt economic activities and infrastructure. These challenges highlight the importance of South Sudan utilising its oil and agricultural sectors for both recovery and strengthening resilience reflecting Pata et al. [59] and Shah et al. emphasis on effective governance in managing energy and environmental resources, given that oil accounts for 90 % of the country's revenue and agriculture is the main source of livelihood for most households [60]. Furthermore, the conflict and economic downturn have worsened poverty levels in South Sudan. From 2009 to 2016, the number of individuals living in poverty rose significantly, triggered by a major economic crisis that led to a decline in the economy because of lower oil revenues and interruptions in production which arein line with the findings of Kolodko [61] on the impact of economic instability on poverty in resource-dependent regions.

### 5.2 Dimensions of the conflict impact

This decline in economic activity has led to widespread displacement and hardships for many, as people's spending has continuously decreased since the beginning of the civil conflict, this a pattern observed in similar contexts [1,56,62]. Inflation has been a major concern, with the South Sudanese currency losing value quickly and causing an increase in the price of imported food items, which has been further exacerbated by market closures and interruptions in trade routes which aligning with Waheed et al. [25] on economic instability and inflationary pressures. The poverty in South Sudan is mainly found in rural areas, where people lack access to essential services, infrastructure, and economic opportunities. The prolonged conflict has greatly damaged the education system, leading to one of the lowest literacy rates in Africa and hindering infrastructure development. This is reflecting Shah et al. (2019) on the adverse impacts of conflict on education and infrastructure. To reduce poverty and boost economic growth, it is recommended that South Sudan end the cycle of violence and address political and macroeconomic risks to create a favourable environment for long-term economic progress as advocated by Yasmeen et al. [28] and Shah et al. [24].

### 5.3 Immediate revenue impacts

The cessation of oil sales has resulted in substantial financial deficits. For South Sudan, the loss is quantified at an estimated 5.7 million USD per day due shutdown of BAPCO pipeline, with approximately 50 % of the government's revenue stream originating from the sales of the Dar blend. Concurrently, the Government of Sudan is confronted with a forfeiture of transportation-related revenues. This includes not only transportation, processing and sovereignty fees, which cumulatively amount to 9.1 USD per barrel but also Transitional Financial Arrangement (TFA) fees, resulting in an approximate daily loss of 865,000 USD (IMF, 2022; [18]). An estimation for direct revenues impact on Sudan and South Sudan has been summaries on Table 4 below.

**Table 4:** Implications of Khartoum War on Sudan and south Sudan.

Cost Type	Entity Affected	Losses Day	Time Frame	Restoration Estimate	Responsible Entity	Long-Term Impact	Comments
**Oil Sales Revenue**	South Sudan	$5.7 million USD	Since disruption commenced	Depending on Sudan infrastructure rehabilitation	South Sudan Government	Decreased national budget	90 % of south Sudan's income a
**Transportation & TFA Fees**	Sudan	$865,000 USD	Since disruption commenced		Sudan Government	Loss in transit revenue	Based on the agreement [18] rates b
**Oil Production Loss (Blocks 1,2,4,6,17)**	Sudan	$2.5 million USD	Since disruption commenced	2–5 years for full infrastructure overhaul	Oil Companies & Environmental Agencies	Compromised energy security, increased reliance on imports, economic destabilization	Estimations based on last production rates c
**Loss of Oil products due to Khartoum Refinery Shutdown**	Sudan and Ethiopia	5.8 Million USD	Since disruption commenced	based on the last production before the war			The agreement with Ethiopia is not active currently[63]
**Infrastructure Deterioration (pipelines and pump stations)**	Sudan	Variable	Ongoing	Variable	Oil Companies	2–5 years for full infrastructure overhaul	The extent of deterioration will require a full assessment post-conflict to determine the complete restoration costs and timelines.
**Khartoum Refinery facilities**	Sudan	Ongoing	Since disruption commenced	2–5 years for full infrastructure overhaul	Khartoum Refinery company	shortage in Supplies of fuel and LGP to a local market	
**leakages and oil contamination**	Sudan	Ongoing	Since disruption commenced	Variable	Oil Companies & Environmental Agencies Oil Companies & Environmental Agencies	Environmental degradation; public health risks	
**Cleaning Contaminated Sites**	Sudan	Variable	Since disruption commenced	Variable			

Notes.

a According to the ministry of petroleum south Sudan [19].

b According to Oil and Related Economic Matters"[18] agreement between Sudan and South Sudan.

c Republic of South Sudan: 2022 Article IV Consultation And Second Review Under The Staff-Monitored Program [60].

### 5.4 Production and operational losses

Production in blocks 1, 2, 4, 6, and 17 has stalled, leading to a loss of about 35,000 barrels per day, equivalent to roughly 2.5 million USD in daily revenue. The ensuing cost implications encompass the maintenance and potential restart of pipelines, alongside the resumption of operations in producing wells, flow lines, trunk lines, and associated facilities.

### 5.5 Infrastructure rehabilitation costs

Damage extends to key infrastructural components, necessitating considerable rehabilitation expenditures to restart and recover these facilities.

### 5.6 Power generation deficits

Less than 65 % of Sudan's population currently has access to electricity, and 40 % of the country's electricity needs are met through fuel-based sources [64,65]. This reliance on fossil fuels makes the impact of the ongoing conflict even more critical for access to electricity, highlighting the vulnerability of energy infrastructure in times of crisis. Exacerbating this situation, The Kosti power plant and the Garri power plant are both experiencing shutdowns due to a shortage of feedstock. The Kosti plant relies on Dar blend and generates around 375 MW of power, while the Garri plant uses liquefied petroleum gas, Petcoke and diesel to produce approximately 450 MW. These shutdowns are having significant impacts on both households and industries.

### 5.7 Petrochemical industries

Before the recent conflict, the Khartoum Refinery played a vital role in the local market by supplying essential raw materials for making plastic products used in packaging, furniture, and other affordable goods in Sudan. The production of key petrochemical products like ethylene and propylene, crucial elements in creating polymers with extensive applications, was a significant contribution of Khartoum Refinery to these industries [43,66]. Moreover, the refinery had provided kerosene and bitumen for the local painting [44]. Although most of the factoeris are in shutdown currently, the shutdown of the Khartoum Refinery can affect vertical integration within the petrochemical sectors in Sudan in the long run. This would lead to local industries losing their primary supplier of raw materials, potentially stalling the growth of the chemical processing industry in the country. The reliance on local petrochemical production underscores the importance of balancing export-oriented growth with domestic industrial needs to maintain the sustainability and development of Sudan's industrial base.

### 5.8 Extended economic and social implications

The war has resulted in a cascade of economic and societal consequences that go well beyond the immediate effect on oil production and infrastructure, similar to the extended effects described by Pathak [67] in conflict scenarios. One of the most notable implications is the continuous managerial expenses at different oil businesses, notwithstanding the halt of activities. This strategy puts a financial burden on businesses without equivalent productivity improvements, putting economic resources at risk that might otherwise be used to mitigate crises or maintain infrastructure, as discussed by Kolodko [61]. Moreover, Some businesses in the sector have already laid off their staff, which worsens the financial struggle of manpower of the sector on the economic toll of conflict-induced layoffs. Moreover, the conflict has led to a scarcity of fuel and a rise in prices in Sudan, posing a threat to the stability of fuel supply in South Sudan; this is consistent with findings by Laville [68] on resource scarcity in conflict zones. These circumstances have the potential to significantly disrupt mechanised agriculture and the industrial sector, causing social and economic consequences as noted by Ahmed and Miller [69]. According to a recent report by the Food and Agriculture Organization (FAO), the ongoing conflicts have precipitated a significant shortage of agricultural inputs, including fuel, which is negatively affecting agricultural production in Sudan [13], mirroring the broader impacts of resource shortages identified by Waheed et al. [25]. The Insufficient fuel supply hinders agricultural machinery, resulting in decreased production of crops, higher food prices, and increased food [69]. Furthermore, the lack of refined oil products interrupts manufacturing cycles and supply systems. Rising operating expenses and lower outputs strain the economy, leading to increased unemployment and poverty in the area. Furthermore, The emigration of highly skilled professionals and researchers from the region contributed to a ‘brain drain’ and depletion of the country's human capital resources [14]. The ongoing conflict in Sudan has had a significant impact on the Corporate Social Responsibility efforts of oil companies, which have focused particularly on the areas of water supply, healthcare, and education for those living near oil fields due to the conflict, difficulties are expected in the maintenance of water infrastructure, causing shortages that affect the daily lives and overall health of local communities [37], this reflecting Garry and Checchi [62] insights on the challenges of maintaining CSR initiatives in conflict zones. Educational initiatives, such as building schools, are typically included in CSR efforts, but have been interrupted by the conflict, impacting the education of children and the future growth of the community.

### 5.9 Environmental impact

The United Nations Environment Programme (UNEP) has observed that at least 40 % of internal conflicts in the past six decades have ties to the exploitation of natural resources [70], such as oil, which is highly relevant to Sudan's situation. The environmental impacts of such conflicts can be profound, with the potential for escalating in the wrong run. Given the challenging accessibility to plants, wells, fields, and pump stations due to the war, there is an increased likelihood of operational disruptions leading to environmental degradation. Leaks from these facilities can result in the contamination of soil and water bodies, endangering local ecosystems and biodiversity. Such incidents not only disrupt the habitats of various species but also compromise the quality of natural resources upon which local communities depend. Furthermore, unplanned shutdowns, especially those not conducted according to environmental safety standards, may lead to the release of hazardous substances and greenhouse gases, exacerbating air and water pollution. cant oil leaks and the unplanned shutdown of oil and gas fields. Moreover, the stop of the Khanoum refinery, which is the main source of cooking gas for household already affected the gas supply. Previously, Cooking gas shortages in Sudan have led to increased trees chop down for fuel; this shift increases deforestation rates, resulting in increased land degradation and desertification. As trees are chopped down for firewood, the soil becomes more exposed and less stable, increasing the chance of desertification, which in turn threatens biodiversity and disturbs local ecosystems [6,71]. This ecological issue is deeply intertwined with socio-economic stability, as competition over dwindling arable land among ethnic groups can inflame conflicts and soil degradation undermines agricultural output, impacting food security [72,73], Thus, energy scarcity is directly linked to regional stability and peace in region. Access to energy correlates with socio-economic development, while its absence can exacerbate conflict and instability. Climate change impacts, notably in the Horn of Africa, intensify these dynamics, making energy access and climate adaptation vital for regional peace [4,5].

As we discussed earlier, considering factors such as oil leaks, lack of energy infrastructure supervision, and changes in fuel consumption behaviour from fossil fuel to wood, we anticipate a long-term negative impact on the environment in Sudan. This situation can be analysed through the lens of the Environmental Kuznets Curve (EKC) hypothesis, which suggests an inverted U-shaped relationship between economic growth and environmental degradation [23,74]. In conflict-affected regions like Sudan and South Sudan, the expected trend towards reduced environmental degradation with economic development is disrupted. The prolonged conflict prevents reaching the EKC's turning point, leading to continuous environmental harm rather than the expected improvement with economic progress [23,75].

## 6 Recommendations

To address the immediate impacts of conflict and prepare for long-term recovery in Sudan and South Sudan, urgent remedial actions and a comprehensive post-war strategy are essential. Immediate actions should focus on stabilizing energy infrastructure through rapid repairs of damaged facilities and the implementation of temporary measures to ensure a continued energy supply. This includes restoring functionality to critical infrastructure components such as pipelines, refineries, and power plants, ensuring that basic energy needs are met to prevent further economic and social disruption. Deploying emergency response teams to contain environmental damage, such as oil spills and contamination, is crucial to mitigating immediate ecological impacts and protecting public health.

Additionally, establishing temporary energy solutions, such as mobile power units or temporary fuel supply chains, can help bridge the gap during infrastructure repairs, specifically for critical sectors like agriculture and food supply chains. Coordination with international relief organisations can facilitate the provision of technical assistance and resources needed for these immediate measures.

For post-war recovery, the strategy should prioritize the reconstruction and modernisation of energy infrastructure with an emphasis on resilience and sustainability. This involves not only rebuilding what was damaged but also upgrading facilities to withstand future conflicts and environmental challenges. Incorporating resilient and sustainable technologies, such as smart grids and advanced monitoring systems, will enhance the reliability and efficiency of energy supply. Diversifying energy sources to include renewable options such as solar, wind, and hydroelectric power will reduce dependency on oil, enhance economic stability, and contribute to environmental sustainability. This diversification should be supported by policies that incentivise renewable energy investments and promote energy efficiency across various sectors.

Comprehensive asset integrity surveys are necessary to evaluate the current state of infrastructure and inform strategic planning and maintenance to ensure long-term operational reliability. These surveys should assess the physical condition of energy facilities, identify potential risks, and prioritize areas for intervention. Implementing a robust maintenance schedule based on these assessments will help prevent future disruptions and prolong the lifespan of infrastructure assets. To protect Sudan and south Sudan from the negative environmental effects of war, it is essential to enhance environmental governance by strengthening regulatory frameworks and building institutional capacity for enforcement. Additionally, conducting environmental risk assessments and developing crisis management plans that include environmental protection measures are crucial for preparedness. International collaboration and support can provide the necessary expertise and funding for environmental restoration efforts. Finally, promoting renewable energy sources and energy efficiency programs can reduce dependence on oil and lower the environmental risks associated with fossil fuel use. These measures collectively aim to build resilience and foster sustainable development in Sudan amidst ongoing conflict.

## 7 Future researches

In the future, researchers should delve deeper into the lasting effects of the conflict on infrastructure and how it influences energy-related actions among the people. It is crucial to find ways to recover and restarting these facilities so they can function safely after the conflict ends. Furthermore, evaluating how well international efforts and policies work in similar situations could offer useful ideas for establishing robust energy systems in conflict-prone areas. Moreover, there is a need for an asset integrity survey to assess the operational reliability and safety of existing infrastructure. This study has the potential to greatly aid in the strategic planning needed to promote sustainable development and peace in regions affected by conflict.

## 8 Conclusions

The paper highlights the significant effects of the Khartoum war on the oil and energy industries in Sudan and South Sudan. It not only points out the immediate disruptions to oil production but also discusses the broader economic, environmental, and humanitarian impacts. Both countries heavily rely on oil and gas, making them susceptible to political unrest and conflict. Therefore, it is crucial to implement strong regional and national strategies to safeguard energy infrastructure and develop emergency plans for facility shutdowns in times of crisis. Additionally, the study stresses the importance of diversifying energy sources and revenue streams towards more sustainable options.

## Data availability

The authors confirm that the data supporting the findings of this study are available within the article or its supplementary material.

## Funding

This research was not funded by any grants

## CRediT authorship contribution statement

**Mohamed Elnourani:** Writing – review & editing, Writing – original draft, Visualization, Validation, Supervision, Software, Project administration, Methodology, Formal analysis, Data curation, Conceptualization. **Hamid Suliman Hamid Elhag:** Writing – review & editing, Writing – original draft, Validation, Methodology, Formal analysis, Data curation, Conceptualization. **Waleed Isameldin Alasad:** Writing – review & editing, Writing – original draft, Validation, Methodology, Investigation. **Mohamed Nasr Bashier:** Writing – review & editing, Writing – original draft, Validation, Methodology, Investigation.

## Declaration of competing interest

The authors declare the following financial interests/personal relationships which may be considered as potential competing interests:

Waleed Isameldin Alasad reports a relationship with Sudanese Ministry of Energy and Petroleum that includes: employment. If there are other authors, they declare that they have no known competing financial interests or personal relationships that could have appeared to influence the work reported in this paper.

## References

[1] UNDP (2023). THE ARAB STATES REGION.

[2] Verhoeven H. (2011).

[3] Patey L.A. (2010). Crude days ahead? OIL and the resource curse in Sudan. Afr. Aff..

[4] Gavin M.D. (2022).

[5] IEA (2022). Africa energy outlook 2022. https://www.iea.org/reports/africa-energy-outlook-2022.

[6] Mertz O., D'Haen S., Maiga A., Moussa I.B., Barbier B., Diouf A., Diallo D., Da E.D., Dabi D. (2012). Climate variability and environmental stress in the Sudan-Sahel zone of West Africa. Ambio.

[7] UNDP (2022). https://www.undp.org/sudan/publications/empowering-sudan-renewable-energy-addressing-poverty-development.

[8] Omer A.M. (2015). Evaluation of sustainable development and environmentally friendly energy systems: case of Sudan. E3 Journal of Environmental Research and Management.

[9] Omer A.M. (2007). Renewable energy resources for electricity generation in Sudan. Renew. Sustain. Energy Rev..

[10] Mohammed Y.S., Bashir N., Mustafa M.W. (2015). Overuse of wood-based bioenergy in selected sub-Saharan Africa countries: review of unconstructive challenges and suggestions. J. Clean. Prod..

[11] Eldowma I.A., Zhang G.X., Su B. (2023). The nexus between electricity consumption, carbon dioxide emissions, and economic growth in Sudan (1971-2019). Energy Pol..

[12] UNEP (2016). *Empowering People to Protect the planet* (UN environment. https://www.unep.org/resources/unep-annual-report-2016.

[13] FAO (2024). https://www.fao.org/documents/card/en?details=CD0053EN.

[14] Hassan M.H.A. (2023). Sudan's disastrous war — and the science it is imperilling. Springer Nature.

[15] UNOCHA (2023). Sudan situation report, 25 September 2023. https://www.unocha.org/publications/report/sudan/sudan-situation-report-25-september-2023-enar.

[16] Lucente A. (2024). Al-MonitorSouth Sudan's oil at risk due to Sudan civil warAl-MonitorSouth Sudan's oil at risk due to Sudan civil war. https://www.al-monitor.com/originals/2024/03/south-sudans-oil-risk-due-sudan-civil-war#ixzz8XXHJsjkP.

[17] Mitchell C. (2023). https://www.spglobal.com/commodityinsights/en/market-insights/latest-news/oil/041723-sudan-fighting-prompts-fears-oil-supply-could-be-affected.

[18] Sudan (2012). South Sudan agreement on oil and related economic Matters. https://peacemaker.un.org/sudan-southsudan-agreement-oil2012.

[19] Petroleum M.o. (2024). Production & entitlement. https://www.mop.gov.ss/monthly-prod.

[20] Le Billon P., Savage E. (2016). Binding pipelines? Oil, armed conflicts, and economic rationales for peace in the two Sudans. African Geographical Review.

[21] Schillinger J., Özerol G., Heldeweg M. (2022). A social-ecological systems perspective on the impacts of armed conflict on water resources management: case studies from the Middle East. Geoforum.

[22] Mutumba G.S., Odongo T., Okurut N.F., Bagire V. (2021). A survey of literature on energy consumption and economic growth. Energy Rep..

[23] Pata U.K. (2021). Renewable and non-renewable energy consumption, economic complexity, CO(2) emissions, and ecological footprint in the USA: testing the EKC hypothesis with a structural break. Environ. Sci. Pollut. Res. Int..

[24] Shah W.U.H., Hao G., Yan H., Zhu N., Yasmeen R., Dincă G. (2024). Role of renewable, non-renewable energy consumption and carbon emission in energy efficiency and productivity change: evidence from G20 economies. Geosci. Front..

[25] Waheed R., Sarwar S., Wei C. (2019). The survey of economic growth, energy consumption and carbon emission. Energy Rep..

[26] Amin A., Altinoz B., Dogan E. (2020). Analyzing the determinants of carbon emissions from transportation in European countries: the role of renewable energy and urbanization. Clean Technol. Environ. Policy.

[27] Saidi K., Omri A. (2020). The impact of renewable energy on carbon emissions and economic growth in 15 major renewable energy-consuming countries. Environ. Res..

[28] Yasmeen R., Zhang X., Tao R., Shah W.U.H. (2023). The impact of green technology, environmental tax and natural resources on energy efficiency and productivity: perspective of OECD Rule of Law. Energy Rep..

[29] Meaza H., Ghebreyohannes T., Nyssen J., Tesfamariam Z., Demissie B., Poesen J., Gebrehiwot M., Weldemichel T.G., Deckers S., Gidey D.G. (2024). Managing the environmental impacts of war: what can be learned from conflict-vulnerable communities?. Sci. Total Environ..

[30] Sowers J.L., Weinthal E., Zawahri N. (2017). Targeting environmental infrastructures, international law, and civilians in the new Middle Eastern wars. Secur. Dialog..

[31] Lacher W., Kumetat D. (2011). The security of energy infrastructure and supply in North Africa: hydrocarbons and renewable energies in comparative perspective. Energy Pol..

[32] Al-Shammari A.M. (2016). Environmental pollution associated to conflicts in Iraq and related health problems. Rev. Environ. Health.

[33] Laffitte T., Moshenets I. (2023).

[34] Saeed I.M., Ramli A.T., Saleh M.A. (2016). Assessment of sustainability in energy of Iraq, and achievable opportunities in the long run. Renew. Sustain. Energy Rev..

[35] IEA (2024). The impact of Russia's invasion of Ukraine on global energy markets - spotlight. https://www.iea.org/spotlights/the-impact-of-russia-s-invasion-of-ukraine-on-global-energy-markets.

[36] Binetti M.N. (2023). Rebuilding energy infrastructures and the manufacturing sector in post-conflict countries. Energy Pol..

[37] Abdelsamad A. (2023). http://www.ijisrt.com818.

[38] Al-Jazz A. (2014). The oil of Sudan: challenges and achievements. Kenana Handbook Of Sudan.

[39] El Tuhami S. (2014). Kenana Handbook of Sudan.

[40] Patey L.A. (2007). State rules: oil companies and armed conflict in Sudan. Third World Q..

[41] Omer A., Idris K.M. (2004).

[42] Mohyaldinn M.E. (2014). Hydraulic behaviour of Higleig-Portsudan pipeline at operation and shutdown conditions. J. Eng. Sci. Technol..

[43] Abd Allah I., Eldien B. (2010). Doctoral dissertation.

[44] KRC (2009). Khartoum refinery company official website. http://krcsd.com/English/Index.asp.

[45] Yuvaraj M.A., Satish G. (2020). Comparative study of different pipe geometries using CFD. Mater. Today: Proc..

[46] ORC (2024). Elobeid refinery company LTD. https://orc.sd/about-us.php.

[47] IMF (2020).

[48] Schrijver N. (1997).

[49] Vité S. (2009). Typology of armed conflicts in international humanitarian law: legal concepts and actual situations. Int. Rev. Red Cross.

[50] Ajiya M. (2022). Their Unending Clamour for Secession and a Threat to National Security (October 15, 2022).

[51] Zabyelina Y., Kustova I. (2015). Energy and conflict: security outsourcing in the protection of critical energy infrastructures. Cooperat. Conflict.

[52] Aiyejina A., Chakrabarti D.P., Pilgrim A., Sastry M.K.S. (2011). Wax formation in oil pipelines: a critical review. Int. J. Multiphas. Flow.

[53] Khan F., Yarveisy R., Abbassi R. (2021). Risk-based pipeline integrity management: a road map for the resilient pipelines. Journal of Pipeline Science and Engineering.

[54] Shin S., Lee G., Ahmed U., Lee Y., Na J., Han C. (2018). Risk-based underground pipeline safety management considering corrosion effect. J. Hazard Mater..

[55] Ossai C.I. (2012). Advances in asset management techniques: an overview of corrosion mechanisms and mitigation strategies for oil and gas pipelines. Int. Sch. Res. Notices.

[56] Ngai J.S.-h. (2011). Energy as a human right in armed conflict: a question or universal need, survival, and human dignity. Brook. J. Int'l L..

[57] Zeith A., Giorgou E. (2023). When the lights go out: the protection of energy infrastructure in armed conflict. https://blogs.icrc.org/law-and-policy/2023/04/20/protection-energy-infrastructure-armed-conflict/.

[58] OCHA (2023).

[59] Pata U.K., Erdogan S., Ozcan B. (2023). Evaluating the role of the share and intensity of renewable energy for sustainable development in Germany. J. Clean. Prod..

[60] IMF (2022). Republic of SouthSouth Sudan: 2022 article IV consultation and second review under the staff-monitored program. https://www.imf.org/en/Publications/CR/Issues/2022/08/03/Republic-of-South-Sudan-2022-Article-IV-Consultation-And-Second-Review-Under-The-Staff-521692.

[61] Kolodko G.W. (2021).

[62] Garry S., Checchi F. (2019). Armed conflict and public health: into the 21st century. J. Publ. Health.

[63] OEC (2022). Ethiopia (ETH) and Sudan (SDN) trade. T. O. o. E. Complexity.

[64] Panos E., Densing M., Volkart K. (2016). Access to electricity in the World Energy Council's global energy scenarios: an outlook for developing regions until 2030. Energy Strategy Rev..

[65] Ritchie H., Roser M., Rosado P. (2020). https://ourworldindata.org/co2-and-greenhouse-gas-emissions.

[66] Ali E.E., Balal A.A., Abbas R.A. (2017). Shrinkage of plastic raw materials (a comparative study of mold). JECS.

[67] Pathak S. (2020). Ecological footprints of war: an exploratory assessment of the long-term impact of violent conflicts on national biocapacity from 1962–2009. Journal of Environmental Studies and Sciences.

[68] Laville C. (2021).

[69] Ahmed H., Miller E.E. (2023). Quantifying the economic impact on farmers from agricultural machinery: a case study of farmers in Sudan. World.

[70] UNDP (2024). Conflict and natural resources. https://peacekeeping.un.org/en/conflict-and-natural-resources.

[71] Whitney J. (2019). Lands at Risk in the Third World.

[72] Dutta Gupta T., Madurga Lopez I., Läderach P., Pacillo G. (2021).

[73] Robinson J. (2005). Desertification and disarray: the threats to plant genetic resources of southern Darfur, western Sudan. Plant Genetic Resources.

[74] Pata U.K., Caglar A.E. (2021). Investigating the EKC hypothesis with renewable energy consumption, human capital, globalization and trade openness for China: evidence from augmented ARDL approach with a structural break. Energy.

[75] Shah W.U.H., Hao G., Yan H., Yasmeen R., Lu Y. (2023). Energy efficiency evaluation, changing trends and determinants of energy productivity growth across South Asian countries: SBM-DEA and Malmquist approach. Environ. Sci. Pollut. Res. Int..

