# Supplementary material for: Khartoum War's echoes in oil and energy sectors: Economic and environmental implications for Sudan and South Sudan
Source: Heliyon. 2024 Jul 23;10(15):e34739. doi: 10.1016/j.heliyon.2024.e34739 (PMC11328044; doi:10.1016/j.heliyon.2024.e34739)
Supplement: Multimedia component 1 [file mmc1.pdf]

AGREEMENT ON

TOTAL FEES AND PAYMENT PROCEDURES

Between

THE GOVERNMENT OF THE REPUBLIC OF THE SUDAN

And

CNPC INTERNATIONAL (DAR) LTD

And

PETRONAS CARIGALI NILE LTD

And

SINOPEC INTERNATIONAL PETROLEUM EXPLORATION AND PRODUCTION CORPORATION

And

TRI OCEAN EXPLORATION AND PRODUCTION

TABLE OF CONTENTS

	PREAMBLE	3
1	DEFINITIONS	4
2	ACCESS RIGHTS, DELIVERY AND REDELIVERY	5
3	FINANCIAL ARRANGEMENTS	6
4	INVOICING AND PAYMENT PROCEDURES	9
5	SPECIFIC RIGHTS AND UNDERTAKINGS	10
6	PAYMENTS FOR TRANSIT FEE, PROCESSING FEE, PIPELINE TARIFF AND SPECIAL FEE	12
7	TERM	13
8	SURVIVING OBLIGATION UPON EXPIRY	13
9	GOVERNING LAW AND DISPUTE RESOLUTION	13

AGREEMENT ON TOTAL FEES AND PAYMENT PROCEDURES

This **AGREEMENT** is entered into on [19], **July 2014**, by and between:

THE GOVERNMENT OF REPUBLIC OF THE SUDAN (hereinafter referred to as "**GoS**"), represented by the Ministry of Petroleum; **and**

CNPC INTERNATIONAL (DAR) LTD, a company duly incorporated and existing under the laws of British Virgin Island (hereinafter referred to as "**CIDL**"); **and**

PETRONAS CARIGALI NILE LTD, a company duly incorporated and existing under the laws of Mauritius (hereinafter referred to as "**PCNL**"); **and**

[illegible]NOPEC INTERNATIONAL PETROLEUM EXPLORATION AND PRODUCTION CORPORATION, a company duly incorporated and existing under the laws of the People's Republic of China (hereinafter referred to as "**SIPC**"),; **and**

TRI OCEAN EXPLORATION AND PRODUCTION, a company duly incorporated, having its offices at 35B, Saray El Maadi Tower, Corniche El Nile, El Maadi, P.O. Box 1313, Ciaro Egypt (hereinafter referred to as **Tri Ocean**)

Preamble:

WHEREAS

(A) The GoS has permanent sovereignty over its territory, and accordingly has jurisdiction over the petroleum facilities constructed or installed for the petroleum operations within its territory;.

(B) CIDL, PCNL, SIPC and Tri-Ocean are the Foreign Contractor Parties to the Exploration and Production Sharing Agreement of Block 3/7 (the "**EPSA**") in the Republic of the Sudan (the "**RoS**") and are producing from Blocks 3/7 in the Republic of South Sudan (the "**RSS**");

(C) The Foreign Party Shippers in RSS have been processing and transporting and are continuing to process and transport their respective crude oil volume entitlements from RSS utilizing the facilities located in the RoS;

(D) The Parties have entered into the Memorandum of Understanding (the "**MOU**") for Blocks 3 and 7 Central Processing Facilities and Transportation System Resolutions dated 24th June 2014.

NOW THEREFORE, in consideration of the above premises, the GoS and the Foreign Party Shippers from RSS, in the spirit of mutual respect, wish to record in further details (including process and procedures) in respect of the Total Fees arrangement, in this Agreement and hereby agree as follows.

1 DEFINITIONS

"Agreement" shall mean this Agreement concerning fees for, processing, transit and transportation of Foreign Party Shippers' Oil Entitlement Volumes produced in the Republic of South Sudan as well as special fees for these volumes.

"Arrears" shall mean arrears and other payments from Government due to the Contractor Parties comprising (i) the direct payment of pipeline tariff and processing fees by the Government of the Republic of the South Sudan ("GoRSS") to the Government pursuant to the Agreement on Oil and Related Economic Matters dated 27th September 2012 (the "AOREM") which shall be subject to reconciliation; and (ii) other arrears which shall be specified in the definitive agreements.

"Cost" shall mean the capital and operating expenditures as generally adopted and used in the International Accounting Procedure in Oil and Gas Industry.

"GoS" shall mean the Government of the Republic of Sudan.

"Foreign Party Shippers" shall mean CIDL, PCNL, SIPC and Tri-Ocean

"MOU" shall mean the Memorandum of Understanding on Blocks 3 and 7 .Transportation System signed by and between the Parties dated 24 June 2014.

"Oil Entitlement Volumes" shall mean such oil volumes produced and entitled to be received by the Foreign Party Contractor from Blocks 3/7 in RSS.

"Owner" of Transportation System means prior to 1 April 2012, the Contractor Parties to COPA and effective 1 April 2012theGoS, and the Contractor Parties to COPA.

E. Kmeed

yang

"Parties" shall mean the GoS and the Foreign Party Shippers.

"Party" shall mean GoS or Foreign Party Shippers

"Processing and Transportation Facilities" shall mean the Blocks 3&7 Al Jabalyn Central Processing Facilities ("CPF") and Block 3&7 Transportation System as defined under the Crude Oil Pipeline Agreement for Blocks 3 and7 (COPA).

"Revenue" shall mean the pipeline Tariff of USD 5.50 per barrel and Processing Fee of USD 1.60 per barrel.

"RoS" shall mean the Republic of the Sudan.

"RSS" shall mean the Republic of South Sudan.

2 ACCESS RIGHTS, DELIVERY AND REDELIVERY

2.1 The GoS hereby reaffirms that the Foreign Party Shippers shall have continued access rights to the Processing and Transportation Facilities for the Foreign Party Shippers' Oil Entitlement Volumes produced in Blocks 3&7 in RSS in accordance with this Agreement and the definitive agreements to be developed.

2.2 The Oil Entitlement Volumes delivered at the inlet of the Block 3&7 CPF shall be redelivered at the outlet of the Block 3&7 Transportation System. The redelivery obligations shall be subject to applicable quality and quantity adjustments, fuel oil consumption and processing and transportation operational losses per the existing processing and transportation procedures and practices in these facilities which shall remain effective until the execution of the Amendment to COTA. The detailed facilities service agreement and amendment to the COTA shall be entered into between Foreign Party Shippers and PDOC as operator of the CPF and the Transportation System and such agreement shall be subject to the approval of GoS.

2.3 All Parties hereby agree that the linefill replacement for the Transportation System shall be contributed by the Shippers and users from RSS in proportion to their respective oil entitlement. The Shippers and users from RSS shall therefore have title to the said linefill that they have contributed. PDOC shall develop the detailed procedure including the calculation of the linefill contribution by Shippers and users from RSS and shall be subject to approval of the Government.

2.4 If oil production in the RSS should become technically or economically non-sustainable, the Foreign Party Shippers shall send written notice to the GoSat least sixty (60) days prior to the estimated suspension of deliveries. Following consultation with the GoS, the Foreign Party Shippers may suspend deliveries of the Foreign Party Shippers Oil Entitlement Volumes under this Agreement from those fields where such non-sustainability exists.

2.5 If the operation of the Processing and Transportation Facilities should become technically or economically non-sustainable, the GoS shall send written notice to the Foreign Party Shippers at least sixty (60) days prior to the estimated suspension of deliveries. Following consultation with the Foreign Party Shippers, the GoS may suspend the operation of those facilities where such non-sustainability exists.

2.6 The GoS hereby guarantee that no third party, except any entity authorized to act on behalf of the GoS to charge the Total Fees under this Agreement, shall charge the Foreign Party Shippers for any amounts due for processing and transportation services stipulated in this Agreement.

2.7 The GoS undertakes that no other charges, fees, taxes, imposts or other duties shall be levied on the processing and transportation services for the Oil Entitlement Volumes redelivered to the Foreign Party Shippers and lifted. Miscellaneous fees relating to Port Sudan for lifting and exporting but not relating to processing and transportation services paid or payable by the Foreign Party shippers prior to the date of this Agreement shall continue to be in force.

3 FINANCIAL ARRANGEMENTS

3.1 The formulation of the Parties with respect to the financial arrangement relating to the Total Fees are negotiated by the Parties with consideration the relevant provisions regarding transit fee, processing fee and tariff agreed among the concerned parties. Total Fees is herein defined as (i) the pipeline tariff of Transportation System (ii) the processing fee at the Al Jabalyn Central Processing Facilities (the "CPF"); and (iii) the transit fee;(iv) special fees in relation to the Oil Entitlement Volumes produced from the RSS, and processed, transported and lifted through the CPF and the Transportation System facilities in the RoS.

3.2 Subject to other terms of this Agreement, in consideration of the access

rights herein above mentioned, Foreign Party Shippers shall be charged the following fees:

a. From 21st June 2013 to 20th December 2016, a Total Fee of USD 19.80per bbl which details are as below :

- Pipeline tariff : USD6.50 per bbl
- Processing Fee : USD1.60 per bbl
- Transit Fee : USD4.00per bbl
- Special Fee : USD7.70 per bbl

b. From 21st December 2016 until 20th March 2022 plus five years extension subject to the approval by the Government, a Total Fee of USD 12.10 per bbl which details are as below :

- Pipeline tariff : USD6.50 per bbl
- Processing Fee : USD1.60 per bbl
- Transit Fee : USD4.00 per bbl

c. Fees payable by the Foreign Party Shippers to GoS for the period from 9thJuly 2011 and until 20thJune 2013 at USD 12.10 per bbl have been settled and closed by the Parties.

3.3 Processing fee

3.3.1 Subject to Article 3.3.3, the Foreign Party Shippers shall pay to the GoS a processing fee of one United States Dollar and sixty cents per barrel (USD 1.60/bbl) for the Oil Entitlement Volumes for processing services in the CPF.

3.3.2 The fee stipulated for in Article 3.3.1 above shall apply for all Oil Entitlement Volumes exported and lifted as per the bills of lading effective from 21st June 2013.

3.3.3 GoS shall be entitled to the processing fee for the quantities exported by the Foreign Party Shippers as stated in the bill of lading. The Contractor Parties to the EPSA shall be entitled to recover with priority given to the unrecovered amounts pertaining to the Central Processing Facilities (OPEX and CAPEX) then to recover future CPF costs, by offset from the amounts payable to the GoS as Processing Fee by Foreign Party Shippers. If the amounts payable by the Foreign Party Shippers to the GoS as Processing Fee are not sufficient to recover the unrecovered CPF OPEX and CAPEX and future CPF costs, the GoS shall guarantee to

E. Ahmed

the Contractor Parties full and timely recovery of the unrecovered costs from the amounts payable by the Foreign Parties to the GoS as the Special Fee. From 21st December2016, the GoS shall pay the CPF operation costs. For avoidance of doubt, if the Contractor Parties have any remaining outstanding unrecovered amounts after 21st December 2016, Contractor Parties shall be entitled to continue to recover these unrecovered amounts from the Processing Fee for amounts processed thereafter.

3.4 Pipeline Tariff

3.4.1 Subject to Article 6.1.4, the Foreign Party Shippers shall pay to the GoS Transportation Tariff of Six United States Dollars and Fifty cents per barrel (USD 6.50/bbl) for the Oil Entitlement Volumes for transportation services in the Block 3&7 Transportation System.

3.4.2 The fee stipulated for in Article 3.4.1 above shall apply to Foreign Party Shippers' Oil Entitlement Volumes exported and lifted as per the bills of lading effective from 21st June 2013.

3.4.3 Petrodar Operating Company Ltd (the "PDOC') as the operator of the Transportation System shall on behalf of the Owners of the Transportation System charge pipeline tariff of USD 5.50 per bbl("Tariff") for transporting the Oil Entitlement Volumes through the Transportation System. GoS shall issue the invoice for the Pipeline Tariff defined in 3.4.1 above as follows:

a) the sum of Five United States Dollars and Fifty cents per barrel (USD 5.50/bbl) shall be payable into a bank account specified by the operator of the Transportation System (PDOC).

b) the sum of One United States Dollar per barrel shall be payable into a bank account specified by GoS.

3.5 Transit Fee

3.5.1 Subject to Article 6.1.1, the Foreign Party Shippers shall pay to the GoS a Transit Fee for their Oil Entitlement Volumes exported and lifted as per the bills of lading through the territory of the Republic of the Sudan.

3.5.2 The Fee stipulated for in Article 3.5.1 above shall apply for all Foreign Party Shippers' Oil Entitlement Volumes exported and lifted as per the bills of lading effective from 21st June 2013.

E. [illegible]

3.6 Special Fee

3.6.1 Subject to Article 3.3.3 and Article 6.1.2, the Foreign Party Shippers shall pay to the GoS a Special Fee for the Oil Entitlement Volumes exported and lifted as per the bills of lading through the territory of the Republic of the Sudan.

3.6.2 The Fee stipulated for in Article 3.6.1 above shall apply to Foreign Party Shippers' Oil Entitlement Volumes effective from 21st June 2013 until 20th December 2016.

4 INVOICING AND PAYMENT PROCEDURES

4.1 All payments from the Foreign Party Shippers to the GoS for Total Fees shall be based on the oil volumes redelivered to the Foreign Party Shippers and lifted at the Bashayer II marine terminal onboard vessels at Port Sudan as specified in the respective bills of lading.

4.2 The GoS shall issue four separate invoices, one for the Pipeline Tariff as defined in Article 3.4.1 above, one for the Processing Fee as defined in Article 3.3.1 above, one for Transit Fee as defined in Article 3.5.1 above and one for Special Fees as defined in Article 3.6.1 above, to each of the Foreign Party Shippers in respect of their Oil Entitlement Volumes which have been redelivered and lifted at the marine terminal and a bill of lading has been issued.

4.3 All Total Fees invoices to Foreign Party Shippers shall be in USD and the payment as provided in Article 3 above shall be made within fifteen (15) days following receipt of such invoices. Such payment may be made in any other freely convertible currency as requested by GoS. The rate of exchange to be applied for the conversion of United States Dollars to the currency of payment shall be the mid-point spot rate posted on Reuters or an equivalent service by the Bank of England at or around 12:00 noon GMT on the date the payment is made.

4.4 The Foreign Party Shippers shall make payment of the invoiced amounts as provided in Article 4.1 above to such bank account(s) of the GoS and PDOC (for the Tariff of USD 5.50 per bbl)as the GoS and PDOC may specify in writing from time to time. Payment shall be made by wire transfer. The Foreign Party Shippers shall give notice to the GoS and PDOC by facsimile or other electronic transmission immediately when payment has been made.

4.5 Processing fees, transportation tariffs, transit fees and Special Fees

payments shall be paid in cash by the Foreign Party Shippers subject to the relevant deductions the Parties agree upon in this Agreement and MOU.

4.6 The GoS may receive payments for Processing Fee, Pipeline Tariff (net tariff of USD 1.00 per bbl), Transit Fee and Special Fee in kind upon its request subject to agreement with the Foreign Party Shippers.

5 SPECIFIC RIGHTS AND UNDERTAKINGS

5.1 Specific rights and undertakings of the GoS

5.1.1 If any of the Foreign Party Shippers fail to pay all or any component of the Total Fees, or any part of the amount of any invoice for processing fees, transportation tariffs, transit fees or Special Fee payments, as herein provided, when such amount is due, the GoS shall issue a default notice to such defaulting Foreign Party Shipper(s), and such defaulting Foreign Party Shipper(s) shall remedy the default within fifteen(15)days from the date of receipt of the default notice. If such defaulting Foreign Party Shipper(s) fail/s to remedy the default within the said fifteen (15) days, then liquidated damages of two percent (2%) per annum above the London Interbank Offered Rate (LIBOR) shall accrue on the unpaid amount from the date of expiry of the remedy period given under the default notice until the date of actual payment.

5.1.2 In the event that such defaulting Foreign Party Shipper(s) fail to remedy the defaulted amount plus the liquidated damages charged as provided above, the GoS shall have the right (right of lien) to sell at international market price FOB Port Sudan such quantity of the defaulting Foreign Party Shippers 'Oil Entitlement Volumes as shall be sufficient to pay such defaulted amount.

5.1.3 In the event that any amount realized by the GoS from the sale of the Foreign Party Shippers' Oil Entitlement Volumes under Article 5.1.2 above is in excess of the unpaid fees and liquidated damages owing by the defaulting Foreign Party Shipper(s) under this Agreement, the GoS shall promptly return to the defaulting Foreign Party Shipper(s) the excess amount. In the event that the GoS fails to return the excess amount, the defaulting Foreign Party Shipper(s) shall have the right to deduct the amount from the next payment due to the GoS.

5.1.4 In addition to the exercise of the right of the GoS to lift the crude oil equivalent to the defaulted amount from any defaulting Foreign Party

E. [illegible] yang

Shippers as provided in Article 5.1.2, the GoS reserves the right to suspend processing and transporting the defaulting Foreign Party Shippers' Oil Entitlement Volumes until such time as the indebtedness is paid or thirty (30) days of continued suspension, whichever is earlier.

5.1.5 The GoS shall have the right to terminate this Agreement, upon thirty (30) days prior written notice, if Foreign Party Shipper(s) commit a material breach to this Agreement and such breach is not remedied within thirty (30) days from the date of a notice issued by the GoS to the Foreign Party Shippers in breach to remedy the breach .For purposes of this Agreement, material breach shall include failure to satisfy payments of fees by the Foreign Party Shippers after the Government has exhausted all remedies available to it as per this Article 5.1.2 and Article 5.1.4.

5.1.6 The Foreign Party Shippers shall indemnify and hold harmless the GoS from and against any liability, loss or damage, including litigation expenses, court costs and attorneys' fees, suffered by the GoS, arising directly out of any demand, claim, action, cause of action or suit brought by any person asserting damages due to a shutdown of the Processing and/or Transportation Facilities ordered by the GoS for breach of this Agreement or failure of the Foreign Party Shipper(s) to pay any amount under this Agreement when it falls due.

5.1.7 The GoS shall indemnify and hold harmless the Foreign Party Shippers from and against any liability, loss or damage, including litigation expenses, court costs and attorneys' fees, suffered by the Foreign Party Shippers arising directly out of any demand, claim, action, cause of action or suit brought by any person asserting damages due to a shutdown of the Processing and Transportation Facilities ordered by the GoS in material breach of this agreement unless such shutdown is due to breach of this Agreement or failure of the Foreign Party Shipper(s)_to pay any amount under this Agreement when it falls due.

5.2 Continuing obligations

5.2.1 Suspension or termination shall not relieve the Party in breach from fulfilling any financial payments under this Agreement that have accrued up to the date of termination, including any claims or damages.

6 PAYMENTS FOR TRANSIT FEE, PROCESSING FEE, PIPELINE TARIFF AND SPECIAL FEE

6.1 The Foreign Party Shippers shall take all necessary measures to ensure that all payments due to GoS accruing for the period from 21st June 2013 and up to the date of signature of this Agreement will be settled within fifteen (15) days following the signature of this Agreement. The settlement of these amounts and future payables shall be subject to the following:

6.1.1. Fifty percent (50%) of the total of the Transit Fee due in each invoice shall be deductible for settlement of Arrears specified in the definitive agreements on arrears stipulated for in Article 7 of the MOU.

6.1.2 Fifty Percent (50%) of the total of the Special fee due in each invoice shall be deductible for settlement of Arrears specified in the Definitive Agreements on arrears stipulated for in Article 7 of the MOU but subject to the recovery of the CPF cost as per Article 3.3.3 of this Agreement.

6.1.3 Fifty percent (50%) of the net operating profit (total Revenue less total Costs) GoS is entitled to as a result of the processing of the Foreign Party Shippers' Oil Entitlement Volumes shall be deductible for settlement of Arrears specified in the Definitive Agreements on arrears stipulated for in Article 7 of the MOU. For avoidance of doubt, this arrangement shall not include the net operating profit GoS is entitled to as a result of processing the Oil Entitlement Volumes of GoRSS.

6.1.4 Fifty percent (50%) of the net operating profit (total Revenue less total Costs) GoS is entitled to as a result of the transportation of the Foreign Party Shippers Oil Entitlement Volumes shall be deductible for settlement of Arrears specified in the Definitive Agreements on arrears stipulated for in Article 7 of the MOU. For avoidance of doubt, this arrangement shall not include the net operating profit GoS is entitled to as a result of transporting the Oil Entitlement Volumes of GoRSS.

6.1.5 Fifty percent (50%) of the total Pipeline Tariff agreed herein less the tariff payable to the operator of the Transportation System (USD6.50/bbl less USD/5.50bbl) which is USD 1.00/bbl.

6.2 For avoidance of doubt, the deductions stipulated in Article 6.1 above shall apply solely and exclusively to Foreign Party Shippers which the GoS owes arrears to.

6.3 The Government shall issue a reconciliation statement with each set of

invoices issued according to the bill of lading clarifying the payments and deductions. The Parties shall meet monthly or at the request of either Party to reconcile invoices, payments and deductions.

6.4 Failure to satisfy payments under Article 6.1 above shall entitle GoS to enforce the right of lien as provided for in Article 5.1.2 above.

7 TERM

This Agreement is legally binding on the Parties signing it and is deemed effective from 24th June 2014 and shall remain in force until 20th March 2022 unless extended by the mutual written agreement of the Parties.

8 SURVIVING OBLIGATION UPON EXPIRY

The expiry of this Agreement shall not relieve the Parties from any outstanding obligations accrued prior to the date of expiry.

9 GOVERNING LAW AND DISPUTE RESOLUTION

This Agreement shall be governed and construed in accordance with the law of the Government of the Republic of the Sudan and any dispute hereunder shall first be settled amicably among the Parties, failing such shall be resolved by arbitration in accordance with the Rules of Arbitration of UNCITRAL and the place of arbitration shall be Dubai. The number of arbitrators shall be three (3); one to be appointed by each Party and the two (2) arbitrators so appointed shall appoint the third arbitrator. The language to be used in the arbitral proceedings shall be English. The arbitration award shall be final and binding on the Parties.

[INTENTIONALLY LEFT BLANK]

E. Swad Yang

Executed in Khartoum, on this day the 19th of July, 2014

For and On Behalf of:

____

Government of the Republic of the Sudan
Name: AWAD ELKARIM M. KHAIR
Title: Sec. General

____

CNPC International (Dar) Ltd.
Name: ZHAO DONG
Title: CHAIRMAN

____

PETRONAS Carigali Nile Ltd.
Name: Mohamad Yusof Shahid
Title: Country Chairman

____

SINOPEC International Petroleum Exploration and Production Corporation
Name: YANG QINGWEN
Title: Deputy Manager

____

Tri Ocean Exploration and Production
Name: M. EL ANSARY
Title: Director
