# Supplementary material for: Khartoum War's echoes in oil and energy sectors: Economic and environmental implications for Sudan and South Sudan
Source: Heliyon. 2024 Jul 23;10(15):e34739. doi: 10.1016/j.heliyon.2024.e34739 (PMC11328044; doi:10.1016/j.heliyon.2024.e34739)
Supplement: Multimedia component 2 [file mmc2.pdf]

Agreement

between

The Government of the Republic of South Sudan

and

The Government of the Republic of the Sudan

on

Oil and Related Economic Matters

Addis Ababa, 27 September 2012

TABLE OF CONTENTS

1 DEFINITIONS 4

2 SOVEREIGNTY 4

3 ACCESS RIGHTS, DELIVERY AND REDELIVERY 5

4 FINANCIAL ARRANGEMENTS 6

5 PAYMENT PROCEDURES 8

6 SPECIFIC RIGHTS AND UNDERTAKINGS 9

7 METERING 12

8 QUALITY ADJUSTMENT PROCEDURES 12

9 CROSS BORDER OPERATIONS 13

10 MONITORING 14

11 REPRESENTATIVES 15

12 MUTUAL FORGIVENESS OF CLAIMS OF OIL RELATED ARREARS AND OTHER CLAIMS 16

13 OIL ON OIL TANKER AND MONIES WITHHELD 16

14 SUDAPET 17

15 RESUMPTION OF OIL PRODUCTION, PROCESSING AND TRANSPORTATION 17

16 FORCE MAJEURE 18

17 DATA 18

18 TRANSPARENCY 18

19 AUDITS 19

20 DETAILED AGREEMENTS AND PROCEDURES 19

21 OTHER AGREEMENTS 19

22 TERM 19

23 EXPIRY 20

Preamble:

Affirming their commitment to promoting the future stability and economic viability of both States;

Mindful of the mutual interest of both States to cooperate between themselves and with their neighbours on the basis of respect for each other's sovereignty, territorial integrity and common pursuit of sustainable development and mutual benefit in accordance with international law;

Acknowledging the importance of carrying out oil related operations with due diligence and efficiency and in accordance with international practice and the key role that oil revenues play with respect to their respective economies;

Recognizing the reality of interdependence and shared interest in the oil sector;

Convinced that they should reach decisions with the above reality in mind and with a view to promoting the economic welfare and wellbeing of the peoples of the two States;

The Parties hereby agree as follows:

1 DEFINITIONS

"Agreement" shall mean this Agreement concerning oil and related economic matters.

"GoRSS" shall mean the Government of the Republic of South Sudan.

"GoS" shall mean the Government of the Republic of the Sudan.

"Oil Entitlement Volumes" shall mean such oil volumes that a party to an exploration and production sharing agreement is entitled to receive thereunder.

"Parties" shall mean the GoRSS and the GoS.

"Petroleum Monitoring Committee" shall mean the committee and the sub-committees established in accordance with Article 10.

"Processing and Transportation Facilities" shall mean the GNPOC central processing facility, the GNPOC transportation system, the Petrodar central processing facility and the Petrodar transportation system.

"RoS" shall mean the Republic of the Sudan.

"RSS" shall mean the Republic of South Sudan.

"TFA" shall mean the transitional financial arrangement provided for in Article 4.4.

2 SOVEREIGNTY

2.1 Each State shall have permanent sovereignty over its natural resources located in or underneath its territory, including petroleum resources.

2.2 Each State shall have sovereignty over all petroleum facilities constructed or installed for the petroleum operations within its territory.

2.3 The Parties agree that the territorial principle applies in the petroleum sectors of both States.

3 ACCESS RIGHTS, DELIVERY AND REDELIVERY

3.1 a. The GoS hereby grants the GoRSS access rights to the Processing and Transportation Facilities for the GoRSS Oil Entitlement Volumes in accordance with this Agreement and the agreements to be developed as provided in sub-Article 3.3 below.

b. The right of access for the crude oil produced in Block 5A shall be subject to quality and capacity constraints of the processing facilities and the transportation system and the Khartoum Refinery.

3.2 The GoRSS Oil Entitlement Volumes delivered at the inlet of the GNPOC processing facilities shall be redelivered at the outlet of the GNPOC transportation system. The GoRSS Oil Entitlement Volumes delivered at the inlet of the Petrodar processing facilities shall be redelivered at the outlet of the Petrodar transportation system. The redelivery obligations shall be subject to applicable quality and quantity adjustments, fuel oil consumption and processing and transportation operational losses.

3.3 a. The Parties shall develop processing agreements for the processing of the GoRSS Oil Entitlement Volumes in the GNPOC and Petrodar processing facilities and transportation agreements for the transportation in the GNPOC and Petrodar transportation systems. The agreements shall be compatible with the existing processing and transportation procedures and practices in these facilities and consistent with this Agreement. The Parties shall aim to conclude the agreements within one (1) month after the signing of this Agreement, unless otherwise agreed.

b. The resumption of oil production, processing and transportation as provided for in Article 15 shall not be dependent upon the conclusion of agreements under sub-Article 3.3 a above. Until such processing and transportation agreements have been concluded, existing practices based on the relevant technical provisions as contained in the relevant Crude Oil Transportation Agreements shall apply unless otherwise provided for in this Agreement.

3.4 The Parties shall take all necessary actions to implement the provisions of this Article and other relevant provisions of this Agreement with the relevant operating companies.

3.5 The GoRSS shall provide their proportionate share of the linefill for the

Petrodar and GNPOC transportation systems. At the expiry of this Agreement, this share of the linefill shall be redelivered to the GoRSS at the marine terminal.

3.6 If oil production in the RSS should become technically or economically non-sustainable, the GoRSS shall send written notice to the GoS at least sixty (60) days prior to the estimated suspension of deliveries. Following consultation with the GoS, the GoRSS may suspend deliveries of the GoRSS Oil Entitlement Volumes under this Agreement from those fields where such non-sustainability exists.

3.7 If the operation of the Processing and Transportation Facilities should become technically or economically non-sustainable, the GoS shall send written notice to the GoRSS at least sixty (60) days prior to the estimated suspension of deliveries. Following consultation with the GoRSS, the GoS may suspend the operation of those facilities where such non-sustainability exists.

4 FINANCIAL ARRANGEMENTS

4.1 Processing fees

4.1.1 The GoRSS shall pay to the GoS a processing fee of one United States Dollar and sixty cents per barrel (USD 1.60/bbl) for the GoRSS Oil Entitlement Volumes for processing services in the GNPOC processing facilities.

4.1.2 The GoRSS shall pay to the GoS a processing fee of one United States Dollar and sixty cents per barrel (USD 1.60/bbl) for the GoRSS Oil Entitlement Volumes for processing services in the Petrodar processing facilities.

4.2 Transportation fees

4.2.1 The GoRSS shall pay to the GoS a transportation tariff of eight United States Dollars and forty cents per barrel (USD 8.40/bbl) for the GoRSS Oil Entitlement Volumes for transportation services in the GNPOC transportation facilities.

4.2.2 The GoRSS shall pay to the GoS a transportation tariff of six United States Dollars and fifty cents per barrel (USD 6.50/bbl) for the GoRSS Oil Entitlement Volumes for transportation services in the Petrodar transportation facilities.

4.3 Transit fee

4.3.1 Whereas, during negotiations, the GoS had first indicated a transit fee of USD 6.00/bbl for the foreign transporters of oil through its territory, the GoS has now set that fee at USD 4.00/bbl; and whereas the GoRSS initially indicated that a transit fee of USD 0.63/0.69/bbl would be appropriate but at the end of the negotiations in August 2012 had considered that it would be reasonable that the GoS grants free transit for all the oil produced in the RSS; neither Party has endorsed, or should be regarded as having endorsed, any of the fees quoted or positions taken by the other Party.

However, in view of the special relationship between the RSS and the RoS, the Parties have agreed that the transit fee for the GoRSS Oil Entitlement Volumes shall be United States one dollar (USD 1.00/bbl). This special fee shall not serve as a precedent, nor shall it have any implication for, or prejudice, any arrangements between either State and any third party. Accordingly, nothing in this paragraph, or in any other part of this Agreement, shall be understood to be the GoRSS's consent - express or implied - to the GoS levying of a transit fee of any value on entitlements other than those of the GoRSS. It is for the GoS to agree with foreign transporters on whatever transit fee it deems fit.

4.4 TFA

4.4.1 The GoRSS shall transfer to the GoS a finite sum of three billion and twenty eight million United States Dollars (USD 3.028 Billion), as a transitional financial arrangement, which the GoRSS will pay in accordance with the payment procedures in Article 5 below, on the basis of a rate of fifteen United States Dollars per barrel (USD 15.00/bbl).

4.4.2 In case the finite sum of the TFA as provided in sub-Article 4.4.1 above, by the way of payment provided for in Article 5, has not been fully paid sixty

(60) days prior to the expiry of the term this Agreement as provided in Article 22, the outstanding balance shall be paid within thirty (30) days prior to the date of expiry.

5 PAYMENT PROCEDURES

5.1 All payments from the GoRSS to the GoS for processing fees, transportation tariffs, transit fees and TFA transfers shall be based on the oil volumes redelivered to the GoRSS and lifted at the marine terminal on board vessels at Port Sudan as specified in the respective bills of lading.

5.2 The GoS shall issue two separate invoices, one for processing fees, transportation tariffs and transit fees, and another for TFA, each time the GoRSS Oil Entitlement Volumes have been finally redelivered and lifted at the marine terminal and a bill of lading has been issued. Payment shall be made by the GoRSS within forty (40) days from the date of the bill of lading.

5.3 The GoRSS shall make payment of the invoice amounts due through the Central Bank of South Sudan to such account(s) of the GoS as the Central Bank of Sudan may specify in writing from time to time. Payment shall be made by wire transfer. The GoRSS shall give notice to the GoS by facsimile or other electronic transmission immediately when payment has been made.

5.4 The invoices will be in United States Dollars, but the GoS shall have the right to request payment in equivalent Euro or Pounds Sterling or in any other convertible currency. The rate of exchange to be applied for the conversion of United States Dollars to the currency of payment shall be the spot rate posted on Reuters or an equivalent service by the Bank of England at or around 12:00 noon GMT on the date the payment is made. The GoS shall bear the costs of the payment transfer and the currency conversion.

5.5 Processing fees, transportation tariffs, transit fees and TFA payments shall be paid in cash by the GoRSS.

5.6 The GoS may receive payments for processing fees, transportation tariffs, transit fees and TFA payments in kind upon its request and with the prior written consent of the GoRSS.

6 SPECIFIC RIGHTS AND UNDERTAKINGS

6.1 Specific rights and undertakings of the GoS

6.1.1 If the GoRSS fails to pay all or any part of the amount of any invoice for processing fees, transportation tariffs, transit fees or TFA payments, as herein provided, when such amount is due, the GoS shall issue a default notice to the GoRSS, and the GoRSS shall remedy the default within fifteen (15) working days from the date of receipt of the default notice. If the GoRSS fails to remedy the default within the said fifteen (15) days, then liquidated damages of two (2) % per annum above London Interbank Offered Rate (LIBOR) shall accrue on the unpaid amount from the date of expiry of the remedy period given under the default notice until the date of actual payment.

6.1.2 In the event that the GoRSS fails to remedy the defaulted amount plus the liquidated damages charged as provided above, the GoS shall have the right (right of lien) to sell at international market price FOB Port Sudan such quantity of the GoRSS's Oil Entitlement Volumes as shall be sufficient to pay such defaulted amount.

6.1.3 In the event that any amount realized by the GoS from the sale of the GoRSS Oil Entitlement Volumes is in excess of the indebtedness and liquidated damages owing by the GoRSS under this Agreement, the GoS shall promptly return to the GoRSS the excess amount. In the event that the GoS fails to return the excess amount, the GoRSS shall have the right to deduct the amount from the next payment due to the GoS.

6.1.4 In addition to the exercise of the right of lien as provided in sub-Article 6.1.2, the GoS reserves the right to suspend processing and transporting the GoRSS Oil Entitlement Volumes until such time as the indebtedness is paid. Any suspension of processing and transportation services for the GoRSS shall remain in effect until the GoRSS has remedied the default or until the indebtedness of the GoRSS under this Agreement has been fully satisfied.

6.1.5 If after suspension of the processing and transportation system the GoRSS still failed to pay the amount which fell due and a period of sixty (60) days has elapsed since the suspension of the processing transportation services, the GoS reserves the right to shut down the processing and transportation system until such time as the indebtedness is paid, upon seven (7) working days prior written notice.

6.1.6 The GoS shall have the right to terminate this Agreement, upon seven (7) working days prior written notice, for any one of the following reasons:

a. If the shutdown of the processing and transportation facilities continues for more than sixty (60) days and the GoRSS has failed to remedy the default.

b. If the GoRSS commits a material breach to this Agreement and such breach is not remedied within sixty (60) days from the date of a notice issued by the GoS to the GoRSS to remedy the breach.

6.1.7 The GoS undertakes to the GoRSS that it shall enter into its own agreements with the relevant operating companies with regard to the transfer to these companies of any financial obligations due to them relating to the processing and transportation of the GoRSS Oil Entitlement Volumes in the processing and transportation facilities.

6.1.8 The GoS hereby guarantees that no third party shall charge the GoRSS for any amounts due for processing and transportation services stipulated in this Agreement.

6.1.9 The GoRSS shall indemnify and hold harmless the GoS from and against any liability, loss or damage, including litigation expenses, court costs and attorneys' fees, suffered by the GoS, arising directly or indirectly out of any demand, claim, action, cause of action or suit brought by any person asserting damage to installations or financial loss due to a shutdown of the Processing and Transportation Facilities ordered by the GoS for material breach of this agreement or failure of the GoRSS to pay any amount under this Agreement when it falls due.

6.1.10 The GoS undertakes that no other charges, taxes, imposts or other duties shall be levied on the processing and transportation services for the GoRSS Oil Entitlement Volumes unless expressly provided for in this Agreement.

6.1.11 The GoS confirms that this Agreement prevails over the Act of the RoS entitled "Amendment to the Petroleum Transit and Services Fees Act 2011", and that it shall not enforce the provisions of this Act against the GoRSS.

6.2 Specific rights and undertakings of the GoRSS

6.2.1 In case the processing, transportation and export of the GoRSS Oil Entitlement Volumes has been interrupted or curtailed due to material breach of this Agreement by the GoS, the TFA amount that would otherwise have been due under Article 4 related to the time period of such material breach shall be suspended until the breach is rectified.

6.2.2 In case suspension in accordance with sub-Article 6.2.1 has continued for more than sixty (60) consecutive days, and the GoS has not remedied the material breach within this period, the GoRSS shall thereafter have the right to terminate this Agreement upon seven (7) working days prior written notice.

6.2.3 The GoS shall indemnify and hold harmless the GoRSS from and against any liability, loss or damage, including litigation expenses, court costs and attorneys' fees, suffered by the GoRSS, arising directly or indirectly out of any demand, claim, action, cause of action or suit brought by any person asserting any damage or financial loss arising out of the failure by the GoS to pay or discharge financial obligations on behalf of the GoRSS to operating companies in respect of charges for processing and transportation through facilities in the RoS, or due to a shutdown of the Processing and Transportation Facilities ordered by the GoS in material breach of this Agreement.

6.3 Continuing obligations

6.3.1 Suspension or termination shall not relieve the party in breach from fulfilling any financial payments under this Agreement that have accrued up to the date of termination, including any claims or damages.

7 METERING

7.1 The Parties shall together with the operating companies review and ensure that effective metering facilities necessary to carry through the payment obligations and other provisions of this Agreement are installed in the RSS and the RoS and in operation prior to the resumption of oil production in the RSS.

7.2 The Parties shall together with the operating companies review whether additional or new metering facilities should be installed in order to be compliant with international standards. To the extent that additional metering facilities are required to this effect, the relevant Party shall instruct the relevant operating company to install such facilities. The investment costs for such additional or new metering facilities shall, unless otherwise agreed, be covered by the Party requesting them, and the facilities shall be owned by such Party. The Parties shall agree on the details for the technical implementation of the above.

7.3 The Petroleum Monitoring Committee shall ensure that all metering facilities are regularly tested and calibrated by an independent third party and that proper maintenance and any necessary repairs are carried out.

8 QUALITY ADJUSTMENT PROCEDURES

8.1 The Parties and the operating companies shall, within forty-five (45) days from the signature of this Agreement, review and adopt quality adjustment procedures in accordance with international practice to ensure that appropriate value or volume adjustments are applied to reflect the differences in quality in the crude oil delivered by the users of the GNPOC central processing facility and the GNPOC transportation system into the blended stream in these facilities.

8.2 If the quality adjustment procedures are not adopted within the time limit set forth under sub-Article 8.1, the Petroleum Monitoring Committee shall appoint international experts to assist in developing such procedures within a time limit to be agreed. The procedures that are developed shall be binding upon the Parties.

8.3 Until possible new quality adjustment procedures are adopted, the existing procedures shall apply.

8.4 Each Party shall have access to the results of quality tests of crude oils delivered into the GNPOC central processing facility and the GNPOC transportation system.

8.5 The Petroleum Monitoring Committee shall monitor the application of the quality adjustment procedure provided for in this Article.

9 CROSS BORDER OPERATIONS

9.1 Cross border movement of personnel and equipment

9.1.1 The Parties shall, in co-operation with the operating companies, enter into agreements to facilitate the movement of personnel, equipment and services across the border between the RSS and the RoS, as well as provide security of personnel, in order to enable efficient oil operations within each State.

9.1.2 The Parties shall together with the operating companies establish a Joint Cross Border Cooperation Committee within twenty-one (21) days after the signing of this Agreement with representatives from each Party and from the operating companies.

9.1.3 The Joint Cross Border Cooperation Committee shall ensure the practical implementation of sub-Article 9.1.1.

9.2 Future cross border petroleum reservoirs

9.2.1 In the event that a cross border petroleum reservoir is discovered, the State in which such discovery is identified shall immediately notify the other State in writing.

9.2.2 Upon receipt of such notification, both States shall in good faith discuss the joint appraisal of the discovery. In case it is deemed commercially viable, the two States shall agree on unitization and development of the discovery.

10 MONITORING

10.1 A Petroleum Monitoring Committee shall be established within twenty-one (21) days of the signing of this Agreement. The Petroleum Monitoring Committee shall oversee the implementation of this Agreement, produce regular reports to the Parties including possible recommendations on the improvement of the co-operation in the petroleum sector, ensure the development of any additional required agreements between the Parties and serve as a forum for seeking resolution to concerns and disputes in respect of this Agreement.

10.2 The Petroleum Monitoring Committee shall consist of two representatives appointed by each Party and a chairperson. The chairperson shall be appointed by the African Union Commission after consultation with the Parties. The Chairperson shall not be an individual who has previously worked with or for either of the Parties.

10.3 Decisions of the Petroleum Monitoring Committee shall be by consensus. In the event that the Committee cannot reach consensus, the issue in question shall be referred to the ministers of petroleum of the two States.

10.4 The Petroleum Monitoring Committee shall establish two subcommittees:

a. A technical committee with capacity to monitor operational aspects relevant to petroleum operations in one State which affect the other State.

b. A financial committee with capacity to review the financial issues between the Parties, including the monthly and yearly reports on these issues prepared by the operating companies and by the governments.

10.5 The Petroleum Monitoring Committee may establish any other subcommittees as it deems necessary to carry out its mandate.

10.6 The Parties shall share equally the costs related to the work of the Petroleum Monitoring Committee, with exception of the costs of their own representatives.

10.7 The Petroleum Monitoring Committee shall quarterly, or as often as it deems necessary, review and verify the correctness of the reported exports of the GoRSS Oil Entitlement Volumes from the operating companies and the corresponding invoices prepared by the GoS. To the extent that any incorrect invoicing is identified, this shall be corrected by adjusting the immediate subsequent invoice(s).

11 REPRESENTATIVES

11.1 The GoRSS representatives at metering stations and facilities in the RoS

11.1.1 The GoRSS shall have the right to have two competent representatives at the GNPOC central processing facility and the Petrodar central processing facility, respectively, to monitor the handling of the GoRSS Oil Entitlement Volumes. The representatives shall have full access to the facilities and to all documents relevant for the processing and export of the GoRSS Oil Entitlement Volumes.

11.1.2 The GoRSS shall have the right to have two competent representatives at the marine terminals in Port Sudan to monitor the proper handling of the GoRSS Oil Entitlement Volumes. The representatives shall have full access to the facilities and to all documents relevant for the storage and loading of the GoRSS Oil Entitlement Volumes.

11.1.3 The GoRSS shall have the right to have two competent representatives at each of the pumping stations in the RoS of the GNPOC Transportation System and of the Petrodar Transportation System to monitor the proper handling of GoRSS Oil Entitlement Volumes. The representatives shall have full access to the facilities and to all documents relevant for the transportation of the GoRSS Oil Entitlement Volumes.

11.1.4 The GoRSS shall have the right to have two competent representatives at any metering station in the RoS that is relevant to oil volumes being processed in or transported through the RoS. The representatives shall have full access to the facilities and to all documents relevant for the transportation of the GoRSS Oil Entitlement Volumes.

11.1.5 The appointment of the representatives provided for in this Article is subject to approval by the GoS.

11.2 The GoS representatives at metering stations and facilities in the RSS

11.2.1 The GoS shall have the right to have two competent representatives at any metering station and facility in the RSS that is relevant to oil volumes being processed in or transported through the RoS. With respect to the Petrodar facilities, such representatives shall have access to the field processing facility at Palogue and to relevant metering stations; and with respect to GNPOC, they shall have access to the field processing facilities

and metering stations in the RSS. The representatives shall have full access to the facilities and to all documents relevant for the transportation of the GoRSS Oil Entitlement Volumes.

11.2.2 The appointment of the representatives provided for in this Article is subject to approval by the GoRSS.

12 MUTUAL FORGIVENESS OF CLAIMS OF OIL RELATED ARREARS AND OTHER CLAIMS

12.1 Each Party agrees to unconditionally and irrevocably cancel and forgive any claims of oil related arrears and other oil related financial claims outstanding to the other Party up to the date of this Agreement, including the claims of arrears and other financial claims filed by each Party with the African Union High Level Implementation Panel on Sudan in February, 2012. This forgiveness shall not include proceeds of oil related to the shipment of the Ratna Shradha, which are currently held in the London High Court and oil onboard the vessel ETC ISIS.

12.2 To that end, each Party acknowledges that there shall be no further liability owed to the other Party in respect of such arrears or other financial claims.

12.3 The Parties agree that the provisions of sub-Article 12.1 shall not serve as a bar to any private claimants and the Parties agree to safeguard the rights of private claimants and ensure that they have the right of access to the courts, administrative tribunals and agencies of each State for the purpose of realizing the protection of their rights.

12.4 The Parties agree to take such action as may be necessary, including the establishment of joint committees or any other workable mechanisms, to assist and facilitate the pursuance of claims by citizens or other legal persons of either State, subject to the provisions of the applicable laws in each State.

13 OIL ON OIL TANKER AND MONIES WITHHELD

13.1 The GoS shall forthwith sell the GoRSS cargo of crude oil in the ETC ISIS vessel and transfer all proceeds to the GoRSS.

13.2 The GoS shall direct funds deposited into the High Court in London related to the GoRSS Oil Entitlement Volumes originally discharged to the vessel Ratna Shradha to be released to the GoRSS.

13.3 The GoRSS shall not bring any other claims related to the prior confiscation and diversion of GoRSS Oil Entitlement Volumes, including for the GoRSS Oil Entitlement Volumes previously diverted to the refineries in the RoS, maintained in domestic reserves or otherwise sold.

14 SUDAPET

14.1 As the Parties at the time of signature of this Agreement disagree and reserve their positions with regard to the consequences of the secession of the RSS on Sudapet's participating interests in exploration and production sharing agreements with contract areas located in the RSS, they shall discuss the matter within a period of two (2) months from the signature of this Agreement with the aim to reach an agreement.

15 RESUMPTION OF OIL PRODUCTION, PROCESSING AND TRANSPORTATION

15.1 The GoRSS shall take all necessary measures to resume the production of oil from all fields in the territory of the RSS, and shall within fourteen (14) days of the signature of this Agreement issue an instruction to the oil companies operating in the RSS to re-establish the oil production in the Blocks 1, 2 and 4, and Block 5A, and the Blocks 3 and 7, and transportation through the Petrodar and GNPOC transportation systems. Resumption of production shall take place as soon as technically feasible.

15.2 The GoS shall take all necessary measures to resume the processing and transportation in the RoS of oil produced in the territory of the RSS when resumed, and shall within fourteen (14) days of the signature of this Agreement issue an instruction to the oil companies operating in the RoS to re-establish both the GNPOC and Petrodar processing facilities and the transportation systems to receive, process and transport such oil. Resumption of the processing and transportation shall take place as soon as technically feasible.

16 FORCE MAJEURE

16.1 Any failure by the Parties in the execution of any of the obligations under this Agreement that is due to a Force Majeure event shall not be considered a breach of the obligation. The Party which cannot fulfil its obligations due to a Force Majeure event shall notify the other thereof and of the details of the Force Majeure event.

16.2 The obligations of the Parties under this Agreement shall be suspended during the time of Force Majeure and the necessary time period thereafter for rectifying any damages caused by the Force Majeure event.

16.3 The Parties shall agree upon the content of a Force Majeure event in the agreements to be developed in accordance with sub-Article 3.3.

17 DATA

17.1 All data, documents and information (including but not limited to geological cores and samples) relevant to the contract areas and petroleum operations in the RSS and in the possession or control of the GoS shall be transferred to the GoRSS and shall be the property of the GoRSS within six (6) months upon the signing of this Agreement unless otherwise agreed.

17.2 The Parties acknowledge that the confidentiality undertakings by the oil companies with the GoS are deemed to have ceased with respect to the contract areas and petroleum operations in RSS at the time of secession.

18 TRANSPARENCY

18.1 The Parties undertake to maintain full mutual transparency of all information relevant to the petroleum activities within the State of one Party that is of relevance for or affects the petroleum activities within the State of the other Party.

19 AUDITS

19.1 The processing and transportation agreements to be developed by the Parties in sub-Article 3.3 above shall provide for audit rights consistent with this Article.

19.2 The agreements referred to in sub-Article 19.1 shall include provisions which establish: (1) a right to the GoRSS to appoint an independent auditor to carry out audits of the books, accounts and records of the operating companies; (2) the confidentiality of the audit process; (3) a process for resolving any discrepancies arising out of such audits; and (4) that expenses in connection with such audits shall be borne by the GoRSS.

20 DETAILED AGREEMENTS AND PROCEDURES

20.1 The Parties shall cooperate and assign the necessary technical and legal personnel in order to develop additional agreements and procedures required for the implementation of this Agreement in an efficient manner.

21 OTHER AGREEMENTS

21.1 The Parties may agree on the purchase by the GoS of any GoRSS Oil Entitlement Volumes at terms to be agreed.

21.2 The Parties may agree on the purchase by the GoRSS of refined products from the GoS at terms to be agreed.

21.3 The Parties may agree on the refining of the GoRSS Oil Entitlement Volumes in the refineries in the RoS at terms to be agreed.

22 TERM

22.1 This Agreement shall remain in force for a period of three (3) years and six (6) months as from the date when the first GoRSS Oil Entitlement Volumes have been redelivered and lifted at the marine terminal and a bill of lading has been issued.

23 EXPIRY

23.1 The expiry of this Agreement shall not relieve either Party from any outstanding obligations accrued prior to the date of expiry.

Done in Addis Ababa, this 27th Day of September, 2012:

....
H.E. Idriss Abdel Gadir
On behalf of the Republic of the Sudan

....
H.E. Pagan Amum Okiech
On behalf of the Republic of South Sudan

Witnessed by:

....
H.E. Thabo Mvuyelwa Mbeki
Chairperson, African Union High Level Implementation Panel
On behalf of the AUHIP
