# Supplementary material for: Khartoum War's echoes in oil and energy sectors: Economic and environmental implications for Sudan and South Sudan
Source: Heliyon. 2024 Jul 23;10(15):e34739. doi: 10.1016/j.heliyon.2024.e34739 (PMC11328044; doi:10.1016/j.heliyon.2024.e34739)
Supplement: Multimedia component 3 [file mmc3.pdf]

بسم الله الرحمن الرحيم

جمهورية السودان

Republic of the Sudan

وزارة الطاقة و النفط

Ministry of Energy & Petroleum

The Minister

الـــوزير

16TH March 2024 **Ref: MEP/M/EX/64**

To: DR. WANG GUOLIN
Director
CNPC International Dar Ltd

MDM. MAI GADALLA
Director
PETRONAS Carigali Nile Ltd

MR. TIAN JIANBO
Managing Director
SINOPEC International Exploration and Production Corporation

Mr. Hassan Meselhy
A. Managing Director
SSTO

SUBJECT: DECLARATION OF FORCE MAJEURE

Reference is made to Article 5.3 of the Agreement on Total Fees for Crude Oil Produced in Blocks 3&7 in South Sudan. Reference is also made to our letter to GoRSS REF: MEP/M/EX/58 dated 14 February 2024.

GoS would like to hereby convey that on 10 February 2024, a restriction of flow was witnessed by the Operator of the Jabalayn-Port Sudan Pipeline (BAPCO) in Pumps Station 5. It became subsequently clear that a gelling incident took place in the area between Pump Station 4 and Pump Station 5. The said area is a military operations zone which has been inaccessible to the Operator (BAPCO) which was, due to the war, unable to supply PS4 with diesel necessary for heating the crude oil to prevent gelling. Ambient temperature in the area in the preceding days was 15ºC indicating a lower ground temperature which amplified the risk of gelling. Additionally, there was no communication with PS4 due to the unavailability of telecommunications in Sudan which were shutdown by RSF in an act of war.

BAPCO subsequently cleared out the gelling and flow resumed only to stop the next day detected by a severe drop in pressure. It was investigated and subsequently discovered that a major rupture took place 18km north of Pump Station 3, and this area is also a military operations area. Teams were mobilized to the area for repair of the rupture and only managed to commence repairs after security clearance was obtained.

Mud pumps received from DPOC in South Sudan were sent to the area between PS4 and PS5 to pump any resistant cold crude oil columns which have gelled in the Transportation System, to allow flow of the crude oil.

There also remains the issue of restart ability of the Transportation System which requires all the pumping stations and heating stations to be fully functional and diesel supply to be adequate and amply available and the gelling issue to be resolved. The resolution of these issues is challenged by the current war conditions in Sudan.

As such GoS is declaring a force majeure which disables us from meeting our obligations in delivering Crude Oil in and through the BAPCO Transportation System to the Bashayer 2 Marine Terminal. We remain ceased to restore the BAPCO Transportation System to operational mode and you will be updated of any developments duly.

We also aspire that you will continue to extend the necessary support to GoS and BAPCO until the operation of the system is restored.

Please accept assurances of my highest considerations

16/3/2024

DR. MOHIELDIN NAIM MOHAMED SAID
Minister of Energy and Petroleum
Republic of the Sudan
